# Autologous haematopoietic stem cell transplantation stabilizes retinal atrophy in multiple sclerosis

**DOI:** 10.1093/braincomms/fcag336

**Published:** 2026-09-02

**Authors:** Jay R T Zoellin, Dario E Mattle, Daniel Agostino, Fabienne C Fierz, Josefine Ruder, Angela Zaugg, Marina Herwerth, Michael Weller, Shiv Saidha, Sven Schippling, Ilijas Jelcic, Patrick Roth, Roland Martin, Veronika Kana

**Affiliations:** Department of Neurology, University Hospital Zurich, Zurich 8091, Switzerland; Institute of Molecular and Clinical Ophthalmology Basel (IOB), Basel 4031, Switzerland; Department of Neurology, University Hospital Zurich, Zurich 8091, Switzerland; Department of Neurology, University Hospital Zurich, Zurich 8091, Switzerland; Department of Ophthalmology, University Hospital Zurich, Zurich 8091, Switzerland; Department of Neurology, University Hospital Zurich, Zurich 8091, Switzerland; Department of Neurology, University Hospital Zurich, Zurich 8091, Switzerland; Department of Neurology, University Hospital Zurich, Zurich 8091, Switzerland; Institute of Pharmacology and Toxicology, University of Zurich, Zurich 8057, Switzerland; Neuroscience Center Zurich, University of Zurich and ETH Zurich, Zurich 8057, Switzerland; Department of Neurology, University Hospital Zurich, Zurich 8091, Switzerland; Neuroscience Center Zurich, University of Zurich and ETH Zurich, Zurich 8057, Switzerland; Division of Neuroimmunology and Neurological Infections, Department of Neurology, Johns Hopkins University School of Medicine, Baltimore, MD 21287, USA; Department of Neurology, University Hospital Zurich, Zurich 8091, Switzerland; Roche Pharma Research and Early Development, Neuroscience and Rare Diseases, Roche Innovation Center, 4070 Basel, Switzerland; Department of Neurology, University Hospital Zurich, Zurich 8091, Switzerland; Department of Neurology, University Hospital Zurich, Zurich 8091, Switzerland; Neuroscience Center Zurich, University of Zurich and ETH Zurich, Zurich 8057, Switzerland; Department of Neurology, University Hospital Zurich, Zurich 8091, Switzerland; Institute of Experimental Immunology, University of Zurich, Zurich 8057, Switzerland; Department of Neurology, University Hospital Zurich, Zurich 8091, Switzerland

**Keywords:** multiple sclerosis, autologous haematopoietic stem cell transplantation, progression, optical coherence tomography, retinal atrophy

## Abstract

Autologous haematopoietic stem cell transplantation is an effective treatment for aggressive multiple sclerosis refractory to disease-modifying therapies. Yet, its impact on neurodegeneration remains underexplored, and few predictive biomarkers for clinical outcomes after autologous haematopoietic stem cell transplantation exist. We investigated whether autologous haematopoietic stem cell transplantation attenuates retinal neurodegeneration and whether optical coherence tomography-derived retinal measures predict disability progression after transplantation. In this single-centre longitudinal cohort study, optical coherence tomography was performed in people with multiple sclerosis treated with autologous haematopoietic stem cell transplantation and non-transplanted controls with relapsing-remitting multiple sclerosis. Retinal layer atrophy rates pre-transplantation and up to 36 months post-transplantation were estimated using linear mixed-effects models, and post-transplantation rates were compared with those of non-transplanted controls. Cumulative link mixed models were used to assess whether baseline retinal layer thickness predicted clinical progression after transplantation. The autologous haematopoietic stem cell transplantation cohort included 39 participants [23/39 (59%) female], comprising 23 with relapsing-remitting [15/23 (65%) female], 8 with secondary progressive [5/8 (62.5%) female] and 8 with primary progressive multiple sclerosis [3/8 (37.5%) female]. The relapsing-remitting multiple sclerosis control cohort on disease-modifying treatment included 48 participants [31/48 (65%) female]. In relapsing-remitting multiple sclerosis, autologous haematopoietic stem cell transplantation reduced thinning of the ganglion cell/inner plexiform layer by 0.65 µm/year (95% CI 0.16 to 1.15, *P* = 0.010), temporal-quadrant peripapillary retinal-nerve-fibre layer by 1.22 µm/year (95% CI 0.63 to 1.81, *P* < 0.001) and papillomacular bundle peripapillary retinal-nerve-fibre layer by 1.59 µm/year (95% CI 0.89 to 2.29, *P* < 0.001). Post-transplantation atrophy rates of the global, temporal and papillomacular-bundle peripapillary retinal nerve fibre layer were lower in the autologous haematopoietic stem cell transplantation cohort than in the control cohort by 0.275 µm/year (95% CI 0.048 to 0.502, *P* = 0.014), 0.294 µm/year (95% CI 0.118 to 0.470, *P* = 0.001) and 0.200 µm/year (95% CI 0.043 to 0.357, *P* = 0.015), respectively. Higher baseline inner nuclear layer thickness predicted better disability outcomes after transplantation in both relapsing and progressive multiple sclerosis in exploratory statistical models (*P* < 0.001). In conclusion, retinal optical coherence tomography demonstrated reduced retinal atrophy rates in relapsing-remitting multiple sclerosis after autologous haematopoietic stem cell transplantation. Higher inner nuclear layer thickness before transplantation predicted more favourable neurological outcomes, indicating that the inner nuclear layer, a retinal layer linked to inflammatory multiple sclerosis activity, may help identify patients most likely to benefit from autologous haematopoietic stem cell transplantation.

## Graphical Abstract

**Graphical Abstract:**
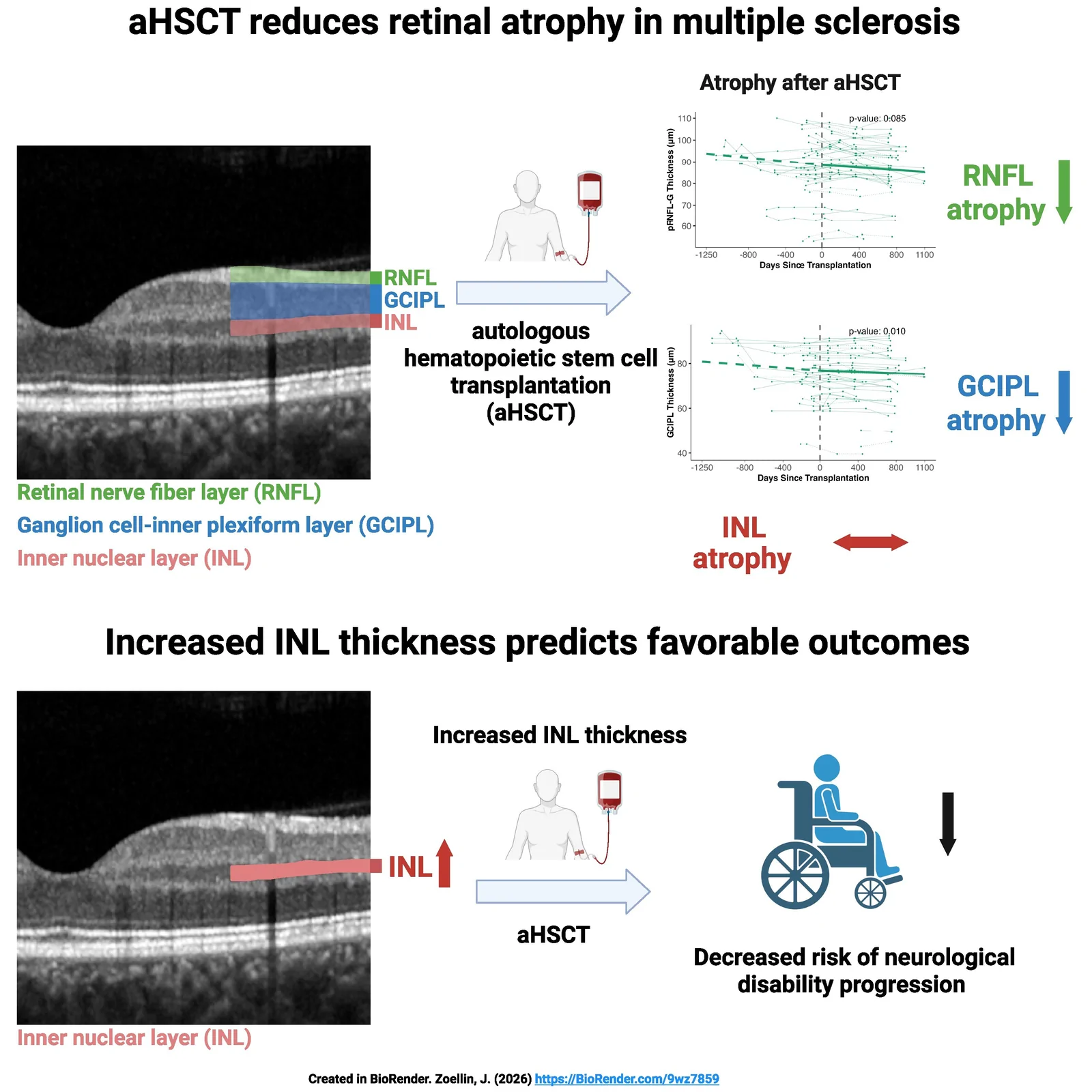

## Teaser

Zoellin *et al*. report that autologous haematopoietic stem cell transplantation is associated with reduced retinal tissue loss in relapsing-remitting multiple sclerosis. Baseline inner nuclear layer thickness predicted more favourable disability outcomes after transplantation, suggesting retinal optical coherence tomography may support monitoring and candie selection.

## Introduction

Multiple sclerosis is a chronic autoimmune disease of the central nervous system characterized by inflammation-driven focal demyelination, gliosis and neurodegeneration.^1,2^ Although typically classified into relapsing and progressive subtypes, accumulating evidence suggests a continuum of disease activity, with progression independent of relapse activity (PIRA) strongly contributing to disability accumulation even in relapsing-remitting multiple sclerosis.^3-7^

Despite the availability of highly effective disease-modifying therapies, some people with multiple sclerosis continue to experience inflammatory activity and worsening of disability.^8^ For these treatment-refractory people with aggressive multiple sclerosis, autologous haematopoietic stem cell transplantation (aHSCT) offers a therapeutic alternative capable of inducing long-term remission.^9,10^ In younger people with relapsing-remitting multiple sclerosis, aHSCT frequently achieves sustained no evidence of disease activity (NEDA-3) for years.^9,10^ However, benefits in progressive multiple sclerosis are more limited,^11^ and whether aHSCT attenuates neurodegeneration remains less clear.^12-15^ Moreover, assessing neurodegeneration by MRI post-aHSCT is initially challenging due to pseudoatrophy, a phenomenon of accelerated brain volume loss immediately after aHSCT.^16-19^ Additionally, due to the toxicity of conditioning regimens, biochemical markers of neurodegeneration transiently increase following aHSCT and inconsistently correlate with disability progression.^12,13,15,16^

Optical coherence tomography (OCT) of the retina provides a non-invasive method for quantifying retinal atrophy in multiple sclerosis.^20-25^ Retinal layers, such as the peripapillary retinal nerve fibre layer (pRNFL) and macular ganglion cell/inner plexiform layer (GCIPL) reflect global central nervous system processes in multiple sclerosis and correlate with disability.^24,26-29^ In people with multiple sclerosis, rates of pRNFL and GCIPL atrophy parallel rates of whole brain atrophy, particularly in cortical grey matter and thalamus.^24,25,27,30^ In contrast, inner nuclear layer (INL) thickness may indicate inflammatory activity.^26,31,32^ While the protective effects of disease-modifying treatments on retinal atrophy are well-documented,^28,33,34^ the impact of aHSCT on retinal atrophy remains understudied.

We therefore investigated whether aHSCT stabilizes retinal atrophy in people with multiple sclerosis using longitudinal OCT imaging. We quantified retinal atrophy before and after aHSCT and compared relapsing-remitting multiple sclerosis participants undergoing aHSCT with an observational relapsing-remitting multiple sclerosis control cohort on disease-modifying treatment. Finally, we examined whether baseline OCT measurements predict long-term disability outcomes following aHSCT.

## Materials and methods

### Study population and cohorts

This single-centre study included people with multiple sclerosis diagnosed according to the 2010^35^ or 2017^36^ revised McDonald criteria. Subtypes were classified as relapsing-remitting multiple sclerosis, secondary progressive multiple sclerosis or primary progressive multiple sclerosis according to the 2013 Lublin definitions.^3^ Two cohorts were analysed: (i) people with highly active multiple sclerosis who underwent aHSCT and were prospectively enrolled in the Swiss aHSCT-in-multiple sclerosis registry (*n* = 39; 23 relapsing-remitting multiple sclerosis, 8 secondary progressive multiple sclerosis, 8 primary progressive multiple sclerosis) and (ii) a moderate-to-severe relapsing-remitting multiple sclerosis observational control cohort from the University Hospital Zurich study (*n* = 53, of whom 48 had OCT data meeting quality criteria and were included in retinal analyses) (both cohorts 2013–2023). Detailed inclusion and exclusion criteria are provided in the Supplementary Methods together with demographic and clinical characteristics of the cohorts (Supplementary Tables 2–4).

### Retinal imaging

In the transplant cohort, OCT imaging was performed 3, 12, 24 and 36 months after transplantation. Before aHSCT, participants were imaged once at a defined timepoint (42–213 days prior to aHSCT) and additionally at irregular intervals prior to aHSCT as part of routine clinical monitoring. In the control cohort, OCT was performed yearly with a Heidelberg Spectralis OCT device (Software version 1.9.10.0; Heidelberg Engineering, Germany) with TruTrack eye-tracking following a standardized protocol^37^ (further details in Supplementary Methods). Eyes were included in the analysis after an optic neuritis (ON)-free interval of at least 6 months. In the case of an ON episode, all scans from the corresponding laterality ranging from 6 months before onset to the end of the observation period were excluded when modelling retinal atrophy rates. When modelling neurological disability progression after aHSCT, scans from all eyes with a documented prior history of an ON episode were excluded based on clinical records (Supplementary Fig. 1).

### Clinical assessments

Neurological disability was monitored using the Expanded Disability Status Scale (EDSS).^38^ In the aHSCT cohort, assessments were performed at defined intervals (baseline, 1, 3, 6, 12, 24, 36, 48 and 60 months after aHSCT, and more frequently if clinically necessary). Prior to transplantation, EDSS scores were recorded once at a defined timepoint (4–292 days prior to aHSCT) and additionally as part of routine clinical monitoring.

### Transplantation protocol

The mobilization, conditioning and transplantation regimens followed the carmustine, etoposide, cytarabine, and melphalan (BEAM)–anti-thymocyte globulin (ATG) protocol.^12,39^ Briefly, cyclophosphamide and G-CSF were used for mobilization, CD34+ haematopoietic stem cells were collected via leukapheresis and autologous CD34+ cells (3–8 × 10^6^/kg) were re-transfused after conditioning.

### Statistical analysis

All statistical analyses and data visualizations were conducted using R (version 4.3.0) and Python (version 3.11.2).^40,41^ Statistical tests were two-sided, with a significance threshold set at an alpha of 0.05. Given the exploratory, hypothesis-generating nature of the study, unadjusted *P*-values are reported and discussed as the primary inferential statistics unless otherwise specified and should be interpreted in light of the number of analyses performed.^28,42^ For the principal analyses presented in the main results tables, Benjamini–Hochberg (BH) correction was additionally applied within each analytical domain, and adjusted *P*-values are reported alongside the unadjusted *P*-values. Further details on statistical tests, model specifications, covariates and additional robustness and sensitivity analyses for the main statistical models are provided in the Supplementary Methods.

Baseline clinical and demographic variables were compared across multiple sclerosis subtypes in the aHSCT cohort using ANOVA, Welch’s ANOVA, Kruskal–Wallis or Wilcoxon tests for continuous or ordinal variables, followed by post hoc pairwise comparisons using Tukey’s Honest Significant Difference test for ANOVA or Dunn’s test with BH corrections for Kruskal–Wallis. Baseline clinical characteristics and demographics in the aHSCT and control cohorts were compared with unpaired Student’s *t*-tests, Welch’s *t*-tests and Wilcoxon rank-sum tests. For categorical variables, chi-square tests with Yates’ continuity correction or Fisher’s exact tests were utilized.

Survival analysis with Kaplan–Meier curves and log-rank tests was used to evaluate NEDA-3 maintenance, with post hoc comparisons employing pairwise log-rank tests with BH corrections. To test for differences in EDSS scores at first and last assessment timepoints, the Wilcoxon signed-rank test was used. Confirmed clinically significant EDSS increase was defined as an increase of ≥1.0 point from a baseline EDSS score ≤5.5, or ≥0.5 points from a baseline EDSS score ≥6.0, confirmed at two subsequent visits.

Longitudinal retinal atrophy was estimated with linear mixed-effects models (LMMs). Consistent with prior studies, data from both eyes of each subject were included in our LMMs. These models estimated atrophy rates while accounting for repeated measurements and inter-eye correlations through random intercepts.^28,43,44^ Atrophy rates were derived from models fitted using restricted maximum likelihood (REML) estimation, with retinal layer thicknesses serving as dependent variables. Atrophy rates were estimated from the fixed effect of time, with group-specific differences derived from interaction terms between time and variable of interest (multiple sclerosis subtype, transplant status or cohort). Model selection was based on the Akaike Information Criterion, Bayesian Information Criterion and marginal and conditional *R*^2^ values from models fitted with ML estimations.^45,46^ Hypothesis testing was conducted using likelihood ratio tests (LRTs) by comparing full models to reduced models lacking a particular fixed effect, both fitted with ML.^47^ To compare retinal layer thickness values at the imaging time points defined in the study protocol (pre-treatment, 3, 12, 24 and 36 months), we excluded all subjects without pre-treatment OCT scans. For each retina, a unique scan (the one closest to the target scan interval (pre-treatment: 40 days before aHSCT, M3: 90 days after aHSCT, M12: 360 days after aHSCT, M24: 720 days after aHSCT, M36: 1080 days after aHSCT) was selected. Retinal layer thicknesses were analysed using LMMs with subject-specific random effects and imaging timepoint as a fixed effect, and hypothesis testing was performed with LRTs.

To link retinal thicknesses on OCT to post-aHSCT disability progression, cumulative link mixed models (CLMMs) with random intercepts, fitted separately for relapsing-remitting multiple sclerosis and progressive multiple sclerosis, were used. OCT images from the last visit before transplantation were retrieved, and the thickness values were averaged across both eyes. In case of a prior ON or insufficient image quality, the contralateral eye was used. Models included age, pre-aHSCT EDSS, years since transplantation, baseline thickness and thickness/time interactions, with longitudinal post-aHSCT EDSS scores being the dependent variable. Effects were reported as odds ratios. Participants were also stratified into above- versus below-mean baseline thickness groups and predicted EDSS trajectories were derived from model-estimated probabilities.

### Standard protocol approvals, registrations and patient consents

This study was approved by the Ethics Committee of the Canton of Zurich, Switzerland (2018 BASEC-No. 2018-01854, BASEC-No. 2013-0001 and BASEC-No. 2020-01187). The institutional USZ Data Governance Board approved this manuscript (Ref. DGR0000811). All patients provided written informed consent for data collection.

## Results

### Study population and retinal imaging

We analysed longitudinal retinal OCT data from 39 people with multiple sclerosis (23 relapsing-remitting multiple sclerosis, 8 secondary progressive multiple sclerosis, 8 primary progressive multiple sclerosis) who underwent aHSCT with the BEAM–ATG regimen,^39^ and 48 people with relapsing-remitting multiple sclerosis on disease-modifying therapies as controls. Study design, inclusion and exclusion criteria, including quality control according to the obvious problems, poor signal strength, centration of scan, algorithm failure, retinal pathology other than MS related, illumination and beam placement (OSCAR-IB) guidelines,^48^ and measurement counts are shown in Supplementary Fig. 1 and Supplementary Table 1. Demographic and clinical characteristics of the cohorts are summarized in Supplementary Tables 2–4. Within the aHSCT cohort, people with relapsing-remitting multiple sclerosis were younger than those with progressive multiple sclerosis forms (*P* < 0.001, Supplementary Table 2) and had lower EDSS scores at the time of transplantation (*P* < 0.001, Supplementary Table 2). The characteristics of the relapsing-remitting multiple sclerosis cohort treated with aHSCT and the control cohort were similar (Supplementary Table 3).

### Clinical outcomes after aHSCT

In people with relapsing-remitting multiple sclerosis, the Kaplan–Meier estimated probability of maintaining NEDA-3 was 95.6% at 12 months post-aHSCT and decreased to 75.2% by 36 months, with all NEDA-3 loss events attributable to disability progression (Supplementary Fig. 2). NEDA-3 maintenance was lower in progressive multiple sclerosis, with a significant difference in time to NEDA-3 loss across subtypes (*P* = 0.015, log-rank test, Supplementary Fig. 2). In the control cohort, the median EDSS score remained stable at 1.0 from baseline to last follow-up (mean follow-up duration: 5.0 years, SD: 2.6 years, *P* = 0.60, Supplementary Table 3). Nevertheless, individual-level analysis showed that 25.0% of participants met the criteria for confirmed clinically significant EDSS increase during follow-up.

### Retinal atrophy rates after aHSCT

Baseline retinal layer thicknesses were similar among multiple sclerosis subtypes before aHSCT (Supplementary Table 5). Importantly, we found no transient changes in absolute retinal layer thickness over time, with no differences in absolute thicknesses of any layer analysed between baseline, 3, 12 and 24 months after aHSCT (Supplementary Fig. 3). Sex, age at aHSCT and time between disease onset and aHSCT were not associated with retinal layer thicknesses in the aHSCT cohort, while a history of ON was associated with lower thicknesses after aHSCT in all layers of the affected eye, except the INL (Supplementary Table 6).

LMMs revealed no differences in atrophy rates between multiple sclerosis subtypes in any retinal layer (Supplementary Fig. 4 and Supplementary Table 7). The estimated GCIPL atrophy rate in relapsing-remitting multiple sclerosis tended to be lower than in progressive subtypes (Supplementary Table 7). Still, the differences did not reach statistical significance even when progressive subtypes were pooled (*P* = 0.995).

### aHSCT stabilizes retinal tissue loss in people with relapsing-remitting multiple sclerosis

**Figure 1:**
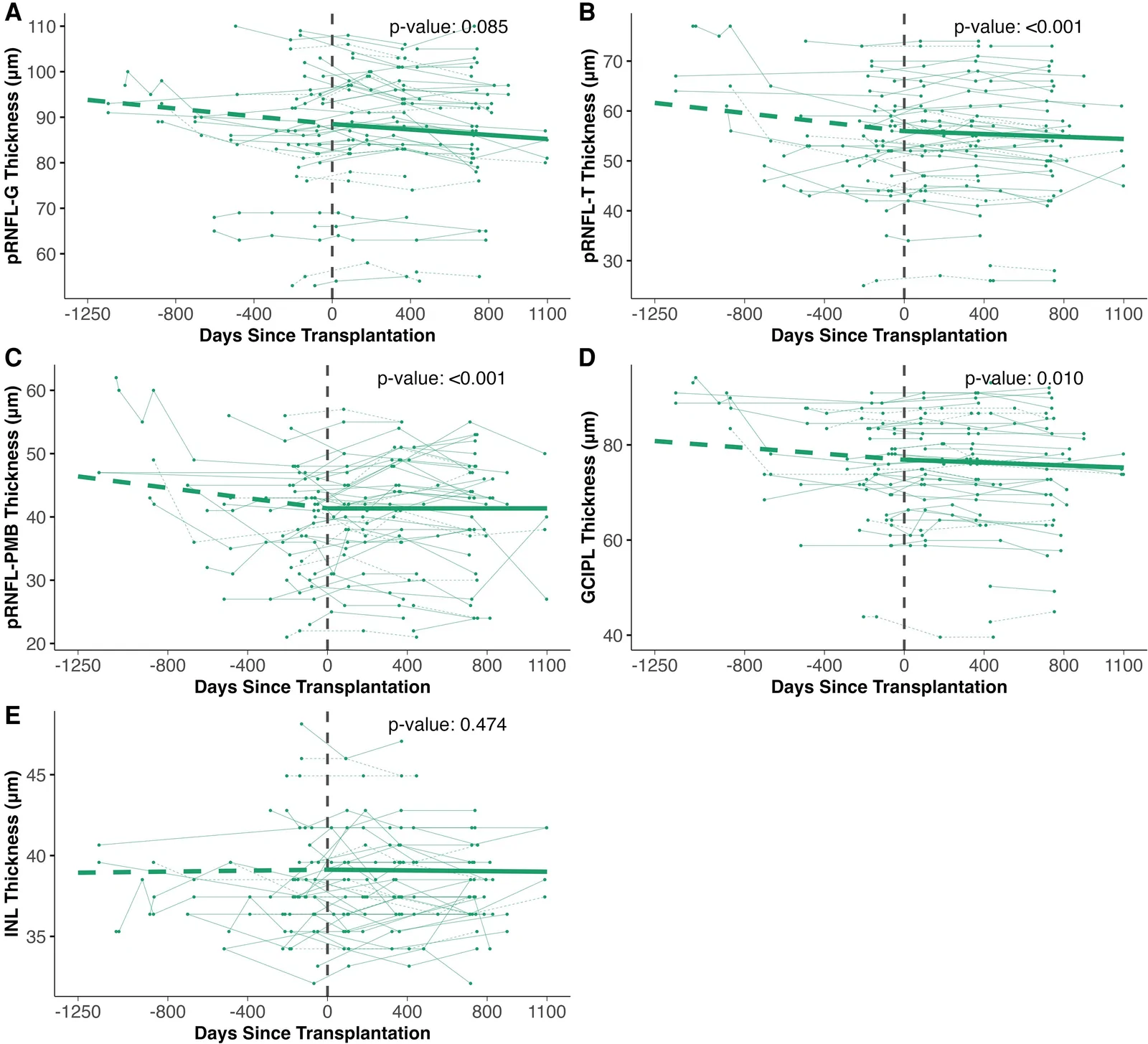
**Retinal atrophy rates before and after autologous haematopoietic stem cell transplantation (aHSCT) in relapsing-remitting multiple sclerosis.** Each panel represents measurements for a different retinal layer: (**A**) Global peripapillary retinal nerve fibre layer (pRNFL-G), (**B**) temporal pRNFL (pRNFL-T), (**C**) papillomacular bundle pRNFL (pRNFL-PMB), (**D**) ganglion cell–inner plexiform layer (GCIPL) and (**E**) inner nuclear layer (INL), showing retinal atrophy before and after aHSCT for relapsing-remitting multiple sclerosis (*n* = 23), with history of optic neuritis (ON) indicated by line shape. Each point represents one eye-level retinal thickness measurement. Fine lines depict progression for individual eyes, while bold lines show model-estimated retinal atrophy rates, computed individually before (dashed) and after aHSCT (solid). The vertical dashed line indicates the day of transplantation, with the intercepts of the bold lines representing the model-estimated retinal thickness at aHSCT. All other fixed effects in the models are not depicted in this graphic, and lines were plotted by taking the estimate of the mode of sex and the median of age at transplantation for intercept adjustment. Hypothesis testing was performed using likelihood ratio tests comparing linear mixed-effects models with and without the pre-/post-aHSCT slope interaction. Test statistics were: pRNFL-G, *χ*^2^(1) = 2.98, *P* = 0.085; pRNFL-T, *χ*^2^(1) = 15.97, *P* < 0.001; pRNFL-PMB, *χ*^2^(1) = 19.18, *P* < 0.001; GCIPL, *χ*^2^(1) = 6.67, *P* = 0.010; and INL, *χ*^2^(1) = 0.51, *P* = 0.474.

Next, we compared atrophy rates before and after aHSCT. Pre-transplant OCT data were available only for people with relapsing-remitting multiple sclerosis. All eligible pre-aHSCT scans (84 macular volume and 90 peripapillary ring scans) and post-aHSCT scans (127 macular volume and 126 ring scans) were used for statistical modelling with LMMs (Fig. 1 and Table 1).

**Table 1:** Estimated retinal atrophy rates before and after aHSCT in relapsing-remitting multiple sclerosis

Retinal layer	pre-aHSCT	post-aHSCT	Difference (post–pre)	*P*-value	BH-adjusted *P*-value
**pRNFL-G, μm/year, mean (SE)**	−1.671 (0.154)	−1.173 (0.252)	0.497 (0.29)	0.085^a^	0.106^a^
**pRNFL-T, μm/year, mean (SE)**	−1.778 (0.160)	−0.561 (0.261)	1.217 (0.3)	<0.001^a^, ^b^	<0.001^a^, ^b^
**pRNFL-PMB, μm/year, mean (SE)**	−1.590 (0.190)	0.001 (0.310)	1.592 (0.357)	<0.001^a^, ^b^	<0.001^a^, ^b^
**GCIPL, μm/year, mean (SE)**	−1.237 (0.137)	−0.586 (0.217)	0.651 (0.252)	**0.010** a, b	**0.0167** a, b
**INL, μm/year, mean (SE)**	0.060 (0.08)	−0.045 (0.127)	−0.105 (0.147)	0.474^a^	0.474^a^

aHSCT, autologous haematopoietic stem cell transplantation; BH, Benjamini–Hochberg; GCIPL, ganglion cell–inner plexiform layer; INL, inner nuclear layer; pRNFL-G, global peripapillary retinal nerve fibre layer; pRNFL-PMB, papillomacular bundle of the peripapillary retinal nerve fibre layer; pRNFL-T, temporal peripapillary retinal nerve fibre layer; SE, standard error.

Difference (post–pre) denotes the difference in atrophy rates (in micrometres/year), where positive values indicate a reduction in tissue loss post-aHSCT compared with pre-aHSCT.

^a^The *P*-values represent the overall significance of the interaction term between transplant status (pre- versus post-aHSCT) and days after transplantation as a predictor of layer thickness changes. These values were obtained using a likelihood ratio test (LRT) to compare two models: a full model including the interaction term for transplant status and days after transplantation and a reduced model that excluded the interaction term. Benjamini–Hochberg adjusted *P*-values are shown in addition.

^b^Bold values represent *P*-values below the threshold of statistical significance at alpha of 0.05.

Following aHSCT, GCIPL atrophy rates were lower by 0.651 µm/year compared with pre-transplantation [95% confidence interval (CI) 0.16 to 1.15, *P* = 0.01, Table 1]. Additionally, transplantation lowered temporal pRNFL (pRNFL-T) and papillomacular bundle pRNFL (pRNFL-PMB) thinning by 1.22 µm/year (95% CI 0.63 to 1.8, *P* < 0.001) and 1.59 µm/year, respectively (95% CI 0.89 to 2.29, *P* < 0.001) in people with relapsing-remitting multiple sclerosis (Table 1). For global pRNFL (pRNFL-G), we observed numerically lower, though not statistically significant, atrophy rates following transplantation, corresponding to a reduction of 0.497 µm/year (95% CI −0.07 to 1.065, *P* = 0.085, Table 1). INL showed no significant post-aHSCT change (−0.105 µm/year, 95% CI −0.39 to 0.18, *P* = 0.474).

Robustness analyses yielded directionally consistent estimates across models adjusting for pre-aHSCT scan timing and after model-based outlier exclusion, with significance preserved for pRNFL-T and pRNFL-PMB and retained or near-significant for GCIPL (Supplementary Fig. 5 and Supplementary Table 8). pRNFL-G and INL reached significance only after outlier exclusion (Supplementary Fig. 5 and Supplementary Table 8).

### Lower retinal atrophy rates with aHSCT versus standard disease-modifying therapy in relapsing-remitting multiple sclerosis

**Figure 2:**
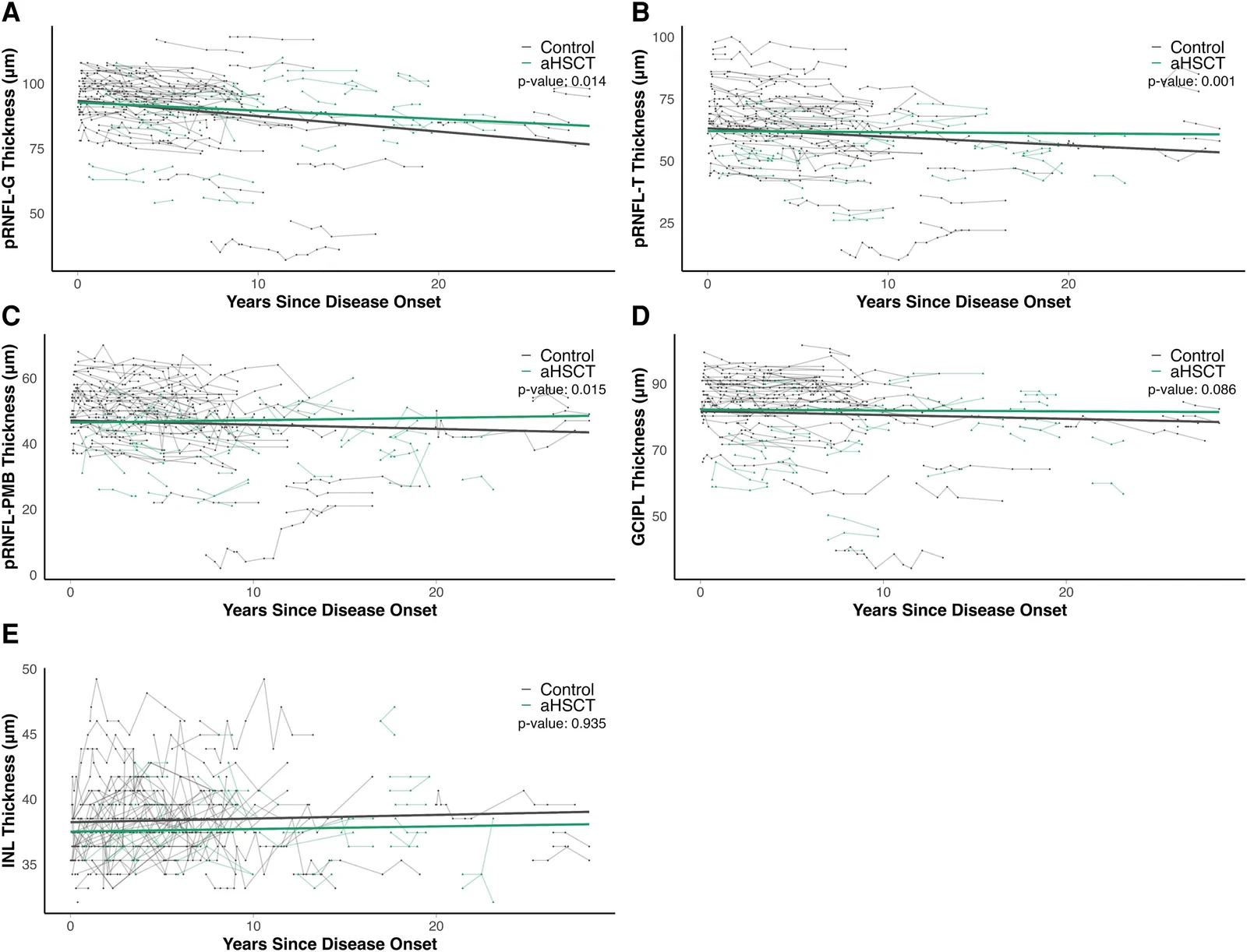
**Longitudinal analysis of retinal layers in transplanted people with relapsing-remitting multiple sclerosis compared with a control relapsing-remitting multiple sclerosis cohort.** Longitudinal analysis of changes in retinal layer thickness over time [Years Since Disease Onset (YSO)] in five retinal layers: (**A**) Global peripapillary retinal nerve fibre layer (pRNFL-G), (**B**) temporal pRNFL (pRNFL-T), (**C**) papillomacular bundle pRNFL (pRNFL-PMB), (**D**) ganglion cell–inner plexiform layer (GCIPL) and (**E**) inner nuclear layer (INL). Each point represents one eye-level retinal thickness measurement. The control cohort is shown in dark grey (*n* = 48), and the transplanted cohort is shown in green (*n* = 23). Individual eye trajectories are shown as lighter lines, while bold trend lines represent cohort-specific estimates derived from linear mixed-effects models. Intercepts for each cohort are adjusted based on the estimates of sex, age at onset, highest ever recorded Expanded Disability Status Scale (EDSS) Score and baseline retinal layer thickness, set to their medians for continuous variables and the mode for categorical variables. The adjustment incorporates the cohort-specific main effect of autologous haematopoietic stem cell transplantation (aHSCT). Slopes represent the interaction of YSO and cohort. The *P*-values were calculated using likelihood ratio tests comparing linear mixed-effects models with and without the cohort-by-YSO interaction. Test statistics were: pRNFL-G, *χ*^2^(1) = 6.04, *P* = 0.014; pRNFL-T, *χ*^2^(1) = 10.76, *P* = 0.001; pRNFL-PMB, *χ*^2^(1) = 5.94, *P* = 0.015; GCIPL, *χ*^2^(1) = 2.96, *P* = 0.086; and INL, *χ*^2^(1) = 0.01, *P* = 0.935.

Retinal atrophy rates of people with relapsing-remitting multiple sclerosis after aHSCT were compared with those of a relapsing-remitting multiple sclerosis reference cohort treated with disease-modifying therapies between 2013 and 2023 (Supplementary Tables 4 and 9). At baseline, the aHSCT cohort had lower mean GCIPL, pRNFL-T and pRNFL-PMB thicknesses (all *P* < 0.001), as well as lower pRNFL-G thickness (*P* = 0.007), than the control cohort (Supplementary Table 9). Compared with the control cohort, transplanted people with relapsing-remitting multiple sclerosis displayed significantly lower baseline-adjusted atrophy rates for pRNFL-G by 0.275 µm/year (95% CI 0.048 to 0.502, *P* = 0.014), for pRNFL-T by 0.294 µm/year (95% CI 0.118 to 0.470, *P* = 0.001) and for pRNFL-PMB by 0.2 µm/year (95% CI 0.043 to 0.357, *P* = 0.015). GCIPL also showed reduced atrophy in the aHSCT cohort, but lacked significance (0.090 µm/year, 95% CI −0.012 to 0.192, *P* = 0.085, Fig. 2 and Table 2).

**Table 2:** Estimated annual atrophy rates in retinal layers for people with relapsing-remitting multiple sclerosis of the control and aHSCT cohorts

Retinal layer	Control relapsing-remitting multiple sclerosis	aHSCT relapsing-remitting multiple sclerosis	Difference (aHSCT-control)	*P*-value	BH-adjusted *P*-value
**pRNFL-G, μm/year, mean (SE)**	−0.593 (0.042)	−0.318 (0.109)	0.275 (0.116)	**0.014** a, b	**0.025** a, b
**pRNFL-T, μm/year, mean (SE)**	−0.342 (0.038)	−0.048 (0.084)	0.294 (0.090)	**0.001** a, b	**0.005** a, b
**pRNFL-PMB, μm/year, mean (SE)**	−0.127 (0.039)	0.073 (0.072)	0.2 (0.080)	**0.015** a, b	**0.025** a, b
**GCIPL, μm/year, mean (SE)**	−0.115 (0.024)	−0.025 (0.047)	0.09 (0.052)	0.085^a^	0.10625^a^
**INL, μm/year, mean (SE)**	0.028 (0.023)	0.02 (0.044)	−0.008 (0.049)	0.935^a^	0.935^a^

aHSCT, autologous haematopoietic stem cell transplantation; BH, Benjamini–Hochberg; GCIPL, ganglion cell–inner plexiform layer; INL, inner nuclear layer; pRNFL-G, global peripapillary retinal nerve fibre layer; pRNFL-PMB, papillomacular bundle of the peripapillary retinal nerve fibre layer; pRNFL-T, temporal peripapillary retinal nerve fibre layer; SE, standard error.

Values represent the estimated annual retinal atrophy rates for the control and aHSCT cohorts, derived from linear mixed-effects models (LMMs). The difference in atrophy rates between cohorts is shown, with positive values indicating reduced tissue loss in the aHSCT group compared with the control cohort.

^a^ *P*-values are derived from comparing the full model (with interaction between years since disease onset and cohort) to the reduced model (without the interaction term) using a likelihood ratio test (LRT) and assessing the statistical significance of the cohort effect on retinal atrophy rates, with Benjamini–Hochberg adjusted *P*-values shown in addition. LMMs were adjusted for years since onset, sex, age at onset, maximum EDSS and baseline retinal layer thickness.

^b^Bold values represent *P*-values below the threshold of statistical significance at alpha of 0.05.

Robustness analyses showed consistent effect directions across alternative model specifications, including baseline-unadjusted and baseline-adjusted models, models with baseline thickness-by-time interaction terms, and analyses restricting control follow-up to narrower years-since-onset windows derived from the aHSCT cohort. Statistical significance was most consistent for the main pRNFL-G and pRNFL-T findings but varied across sensitivity models for GCIPL and pRNFL-PMB (Supplementary Tables 10 and 11 and Supplementary Fig. 6).

### Higher baseline INL thickness is associated with improved neurological outcomes after aHSCT

We employed CLMMs to examine whether baseline retinal layer thicknesses before aHSCT predicted longitudinal EDSS changes. We included baseline OCT measurements before aHSCT (mean: −101 days, min: −213 days, max: −42 days) from 31 people with multiple sclerosis [19 relapsing-remitting multiple sclerosis, 12 progressive multiple sclerosis (7 secondary progressive multiple sclerosis, 5 primary progressive multiple sclerosis)]. EDSS scores were assessed 168 times after transplantation, with an average of 5.4 times per individual (min: 3, max: 8, SD: 1.23). Follow-up intervals for EDSS scores ranged from 166 days to 1795 days after transplantation (average: 910 days).

Higher baseline INL thickness was associated with slower EDSS progression in both relapsing-remitting multiple sclerosis and progressive multiple sclerosis (Table 3). Specifically, each additional micrometre of baseline INL thickness was associated with lower annual odds of being in a higher EDSS category in relapsing-remitting multiple sclerosis (OR = 0.725, *P* < 0.001) and progressive multiple sclerosis (OR = 0.871, *P* < 0.001, Table 3 and Supplementary Table 12). This association was supported by bootstrap analyses in both relapsing-remitting multiple sclerosis (median OR 0.727, 95% CI 0.526 to 0.975) and progressive multiple sclerosis (median OR 0.797, 95% CI 0.333 to 0.927). Leave-one-patient-out (LOPO) analyses showed no direction reversal, with significance retained in 19/19 refits for relapsing-remitting multiple sclerosis and 10/12 refits for progressive multiple sclerosis, although the progressive multiple sclerosis model showed evidence of numerical instability (Supplementary Table 12). Baseline GCIPL thickness in relapsing-remitting multiple sclerosis and baseline pRNFL-PMB thickness in progressive multiple sclerosis were also significantly associated with EDSS outcomes in the primary models (Table 3). However, model diagnostics suggested numerical instability, and these associations were not consistently supported by bootstrap and LOPO robustness analyses. Bootstrap confidence intervals crossed 1, LOPO analyses showed direction reversal and significance was retained in only 4/19 refits for GCIPL in relapsing-remitting multiple sclerosis and 4/12 refits for pRNFL-PMB in progressive multiple sclerosis (Supplementary Table 12).

**Table 3:** Baseline retinal layer thickness as a statistical predictor of neurological disability progression following aHSCT

	Odds ratio (per µm)/bootstrap median (95% CI)		Raw *P*-value/BH-adjusted	
Variable	Relapsing-remitting multiple sclerosis	Progressive multiple sclerosis	Relapsing-remitting multiple sclerosis	Progressive multiple sclerosis
**pRNFL-G (avg)**	1.026/1.021 (0.912 to 1.072)	0.93/0.986 (0.896 to 1.15)	0.155/0.249	0.356/0.398
**pRNFL-T (avg)**	1.04/1.028 (0.896 to 1.142)	1.016/1.021 (0.914 to 1.117)	0.174/0.249	0.398/0.398
**pRNFL-PMB (avg)**	1.037/1.022 (0.812 to 1.195)	1.03/1.037 (0.932 to 1.183)	0.36/0.398	**<0.001/<0.001** a
**GCIPL (avg)**	1.01/1.006 (0.891 to 1.086)	0.982/0.971 (0.849 to 1.109)	**<0.001/<0.001** a	0.162/0.249
**INL (avg)**	0.725/0.727 (0.526 to 0.975)	0.87/0.797 (0.333 to 0.927)	**<0.001/0.00226** a	**<0.001/** **<** **0.001**^a^

Avg, average; BH, Benjamini–Hochberg; CI, confidence interval; EDSS, Expanded Disability Status Scale; GCIPL, ganglion cell–inner plexiform layer; INL, inner nuclear layer; pRNFL-G, global peripapillary retinal nerve fibre layer; pRNFL-T, temporal peripapillary retinal nerve fibre layer; pRNFL-PMB, papillomacular bundle of the peripapillary retinal nerve fibre layer.

Odds ratios represent the effect of each additional micrometre in baseline retinal layer thickness on the annual odds of transitioning to a higher Expanded Disability Status Scale (EDSS) category. Values are derived from cumulative link mixed models (CLMMs), which include a random intercept for each individual and fixed effects for baseline EDSS, age at transplantation, time since aHSCT (in years), baseline retinal thickness and an interaction term between baseline thickness and time. *P*-values reflect likelihood ratio tests (LRTs) comparing the full model (including the interaction) to a reduced model (excluding the interaction), with Benjamini–Hochberg-adjusted *P*-values shown in addition. Significant interactions suggest that the rate of EDSS progression over time depends on baseline retinal layer thickness. Bootstrap values represent patient-level bootstrap median odds ratios with 95% bootstrap confidence intervals based on 500 bootstrap resamples.

^a^Bold values represent *P*-values below the threshold of statistical significance at alpha of 0.05.

When stratifying participants by mean baseline INL thickness, people with relapsing-remitting multiple sclerosis with INL thickness above the group mean (mean INL = 37.95 µm; *n* = 10 above and 9 below the group mean) had 64% lower annual odds of progressing to a higher EDSS category compared with below the group mean (OR = 0.36, *P* = 0.025) (Supplementary Fig. 7). In progressive multiple sclerosis (mean INL = 38.48 µm; *n* = 6 above and 6 below group mean), a nearly 88% reduction was observed (OR = 0.13, *P* < 0.001) (Supplementary Fig. 7). In contrast, when stratifying by mean GCIPL thickness in relapsing-remitting multiple sclerosis or pRNFL-PMB thickness in progressive multiple sclerosis, we found no differences in progression odds between groups (*P *= 0.599 and *P *= 0.344, respectively).

## Discussion

Neurodegeneration is a major driver of long-term disability in multiple sclerosis.^6^ Although aHSCT has emerged as an effective therapy for aggressive, treatment-refractory multiple sclerosis, its long-term effects on neurodegeneration remain insufficiently defined, partly due to challenges in assessing tissue loss immediately after transplantation.^12,49^ Retinal OCT is a biomarker for neurodegeneration, with a large body of literature describing its prognostic value in multiple sclerosis.^20,27,29^ Here, we found decreased retinal atrophy rates in several layers after aHSCT and compared with the control cohort, indicating that aHSCT may reduce retinal neurodegeneration in multiple sclerosis. Additionally, we found that retinal OCT before aHSCT may serve as a prognostic biomarker of long-term disability outcomes.

Brain MRI studies have shown transient increases in brain atrophy after aHSCT, commonly attributed to pseudoatrophy.^17,19,49^ Similarly, serum neurofilament light chain (sNFL) levels rise temporarily in the early post-transplantation period.^12,16^ In contrast, we found no such transient changes in the retina, with layer thicknesses remaining stable in the early months after aHSCT. This highlights the value of retinal OCT as a non-invasive biomarker of neurodegeneration, minimally confounded by pseudoatrophy and thus potentially suited for monitoring neurodegeneration immediately after aHSCT.

A key mechanistic question is whether aHSCT solely reconstitutes adaptive immune function or also suppresses the chronic, compartmentalized inflammation thought to drive PIRA.^6^ Both pRNFL and GCIPL measurements serve as important biomarkers of neurodegeneration in multiple sclerosis.^20^ aHSCT reduced GCIPL, pRNFL-T and pRNFL-PMB thinning in people with relapsing-remitting multiple sclerosis in our cohort. Moreover, atrophy rates for pRNFL-G, pRNFL-T and pRNFL-PMB were significantly lower after aHSCT, despite prior aggressive multiple sclerosis, compared with an observational control cohort receiving intermediate- to high-efficacy disease-modifying therapies. GCIPL atrophy rates were also lower in the transplanted cohort compared with the control cohort, although this difference was not statistically significant. Thus, aHSCT may reduce global neurodegeneration in highly active multiple sclerosis. This aligns with previous studies showing that aHSCT reduces disability accumulation, a process strongly driven by underlying neurodegeneration.^6,9,10^ Further, the late normalization of MRI-derived brain atrophy and sNFL levels after aHSCT supports an aHSCT-mediated reduction in CNS neurodegeneration, consistent with our findings.^12,13,18,49^ Collectively, these results imply that aHSCT’s benefits extend beyond controlling inflammatory relapses and may include slowing of concurrent neurodegenerative processes.

Although GCIPL and pRNFL thicknesses are established predictors of disability progression in people with multiple sclerosis on standard disease-modifying therapies,^20^ this relationship was less consistent after aHSCT. Surprisingly, in our cohort, higher baseline GCIPL thickness in relapsing-remitting multiple sclerosis and higher baseline pRNFL-PMB thickness in progressive multiple sclerosis before aHSCT were associated with worse post-transplantation EDSS outcomes. However, these findings should be interpreted with caution. Their effect sizes were small, and the robustness analyses did not support them. We therefore do not interpret these associations as reliable evidence that greater GCIPL or pRNFL-PMB thickness is associated with increased disability progression after aHSCT. In contrast, baseline INL thickness showed the most consistent and robust association with disability outcomes across both relapsing-remitting multiple sclerosis and progressive multiple sclerosis. Higher INL thickness was associated with lower odds of disability worsening, with supportive bootstrap estimates and no direction reversal in LOPO analyses. Prior studies have associated INL thickness with inflammatory activity in multiple sclerosis, including relapse risk, new contrast-enhancing MRI lesions, new T2 lesions and EDSS progression.^32^ Reductions in INL thickness following initiation of disease-modifying therapies have been linked to inflammatory control and NEDA status, supporting its role as a dynamic biomarker of treatment response.^31^ Our findings extend this evidence, suggesting that greater baseline INL thickness, reflecting inflammatory activity,^26,31,32^ may identify people with multiple sclerosis most likely to benefit from aHSCT although this requires valiion. Our data thus further support selecting people with pronounced inflammatory multiple sclerosis activity as transplantation candies.^9^

Several limitations warrant consideration. First, the number of pre-transplant OCT scans in progressive subtypes was limited, even when pooled, precluding robust pre/post comparisons. In addition, most pre-aHSCT scans in relapsing-remitting multiple sclerosis were obtained from routine clinical monitoring rather than standardized research protocols, and COVID-19 pandemic-related disruptions reduced post-transplant imaging frequency. However, multiple sensitivity analyses addressing pre-aHSCT timing heterogeneity and differential follow-up duration between cohorts did not indicate that these factors substantially altered the main findings. Second, the aHSCT cohort had lower baseline retinal thicknesses than the control cohort. Although we addressed this by statistically adjusting for baseline retinal thickness in the between-cohort models and by performing additional sensitivity analyses, residual concern about a floor-effect-like bias cannot be fully excluded. Third, because individuals selected for aHSCT by indication have highly active treatment-refractory disease prior to transplantation, the observational relapsing-remitting multiple sclerosis control cohort was selected to represent an active moderate-to-severe disease course to avoid comparison with a mild or clinically stable relapsing-remitting multiple sclerosis cohort. Disease-modifying therapy (DMT) stability was therefore not required in the control cohort. Accordingly, residual differences in ongoing disease activity and treatment exposure cannot be excluded. Fourth, the identification of predictive retinal biomarkers was retrospective, and the post-aHSCT CLMM analyses were based on small subtype-stratified samples. Accordingly, these findings should be regarded as exploratory and hypothesis-generating and require confirmation in larger prospective cohorts. Fifth, in the between-cohort analysis, the estimated pRNFL-PMB slope in the aHSCT cohort was slightly positive. Given its small magnitude and lack of consistent robustness, we interpret this cautiously as more likely reflecting residual model sensitivity, regression to the mean or measurement variability rather than true biological thickening. Finally, OCT assesses retinal tissue rather than directly visualizing CNS lesions and thus may only reflect regional processes.^25^ Complementary imaging modalities such as susceptibility-weighted MRI or PET may help localize ongoing pathology more precisely.^50^

In conclusion, our findings indicate that aHSCT can attenuate multiple sclerosis-related retinal atrophy, particularly in people with relapsing-remitting multiple sclerosis, suggesting that it may reduce neurodegeneration in the CNS.^13^ We show that retinal OCT can serve as a robust biomarker of neurodegeneration after aHSCT, potentially avoiding transient confounders such as pseudoatrophy and complementing brain imaging and biochemical markers.^16^ Baseline INL thickness emerged as a potential prognostic marker of long-term disability outcomes, although valiion in larger prospective cohorts is required. Future studies integrating standardized retinal OCT protocols with advanced neuroimaging and molecular biomarkers will be crucial for eluciing the neuroprotective mechanisms of aHSCT and optimizing candie selection for this invasive but highly effective therapy.

## Supplementary Material

fcag336_Supplementary_Data

## Data Availability

The data that support the findings of this study and coded data not published within this article are available from the corresponding author, upon reasonable request, and provided appropriate data transfer agreements. The corresponding author had full access to all study data and takes responsibility for the integrity of the data and the accuracy of the analyses. The code used for the analyses is available at: https://github.com/jayzoellin/Autologous-hematopoietic-stem-cell-transplantation-stabilizes-retinal-atrophy-in-multiple-sclerosis/tree/main.
