# Supplementary material for: Autologous haematopoietic stem cell transplantation stabilizes retinal atrophy in multiple sclerosis
Source: Brain Commun. 2026 Sep 2;8(5):fcag336. doi: 10.1093/braincomms/fcag336 (PMC13563297; doi:10.1093/braincomms/fcag336)
Supplement: Supplementary file 1 [file fcag336_supplementary_data.pdf]

Supplementary material for online publication only

Supplementary Methods

Supplementary References

Supplementary Figures

Supplementary Figure 1 Flowchart of study design for the (A) aHSCT cohort and (B) control cohort.
Supplementary Figure 2 NEDA-3 maintenance after aHSCT by disease subtype.
Supplementary Figure 3 Retinal layer thickness following transplantation.
Supplementary Figure 4 Retinal thickness measurements after transplantation by MS subtype.
Supplementary Figure 5 Effect of pre-aHSCT timing covariates on estimated pre/post retinal atrophy differences in RRMS.
Supplementary Figure 6 Sensitivity analysis for differential follow-up duration between the control and aHSCT cohort.
Supplementary Figure 7 Longitudinal EDSS trajectories following aHSCT by baseline INL.

Supplementary Tables

Supplementary Table 1 Summary of clinical and imaging data in the aHSCT cohort by disease subtype.
Supplementary Table 2 Demographics and clinical characteristics of the aHSCT cohort.
Supplementary Table 3 Demographics and clinical characteristics of people with relapsing-remitting MS in observational control and aHSCT cohorts.
Supplementary Table 4 Disease modifying treatment in relapsing-remitting MS control cohort.
Supplementary Table 5 Baseline retinal layer measurements in the aHSCT cohort.
Supplementary Table 6 Effects of clinical characteristics on retinal thickness in the aHSCT cohort.
Supplementary Table 7 Estimated yearly retinal atrophy by disease subtype following aHSCT.
Supplementary Table 8 Outlier exclusion analyses for the RRMS pre/post retinal atrophy comparisons.
Supplementary Table 9 Baseline retinal layer thickness in the control and aHSCT relapsing-remitting MS cohorts.
Supplementary Table 10 Effect of baseline retinal thickness adjustment on estimated atrophy-rate differences between the aHSCT and control cohorts.
Supplementary Table 11 Characteristics of the restricted control cohorts used in the YSO-window sensitivity analysis.
Supplementary Table 12 Robustness analyses for cumulative link mixed models of post-aHSCT disability progression.

Supplementary Methods

Study Population and Cohorts

This study is a single-center study with retrospective cross-sectional and longitudinal analyses of prospectively collected data from two distinct cohorts of PwMS. For the aHSCT cohort, inclusion criteria were an age between 18 and 55 years, EDSS between 2.0 and 6.5, as well as breakthrough disease activity defined as (1) clinical activity (clinically confirmed MS relapse), (2) MRI activity (contrast-enhancing or new T2-hyperintense lesions on brain or spinal cord MRI), and/or (3) confirmed disability progression based on increases in EDSS, sustained for at least six months, despite treatment with at least one highly effective disease-modifying therapy.[1] Data from 42 people with MS (24 RRMS, 10 SPMS, and 8 PPMS) of the Swiss aHSCT-in MS registry were available, but only 39 (23 RRMS, 8 SPMS, 8 PPMS) were enrolled in this study due to the late enrollment of three individuals in the retinal imaging arm of the study protocol. Our second cohort, termed the observational control cohort, was recruited from a Zurich observational cohort, followed between 2013 and 2023 (BASEC-No. 2013-0001 and BASEC-No. 2020-01187) and consisted of 63 individuals diagnosed with RRMS. Of these 63 participants, 53 fulfilled criteria for an active moderate-to-severe disease course, defined by evidence of clinical and/or radiological disease activity during follow-up, treatment with intermediate- or high-efficacy DMT, and/or escalation to intermediate- or high-efficacy DMT. Ten individuals who did not meet these criteria were classified as having a mild disease course (no clinical or radiological disease activity and treatment with platform therapy or no DMT), and were excluded (Supplementary Fig. 1). Of the 53 clinically eligible individuals, 48 had longitudinal OCT and clinical follow-up data suitable for the present analyses and constituted the final observational RRMS control cohort. DMT stability during follow-up was not required. In the final RRMS control cohort, 36/48 participants had more than one recorded DMT, 34/48 showed escalation to a higher DMT efficacy category, and 27/48 received high-efficacy DMT at some point during follow-up. Confirmed clinically significant EDSS increase occurred in 12/48 participants. DMT exposure and treatment changes are summarized in Supplementary Table 4.

Retinal Imaging

Retinal imaging by OCT included macular volume scans and peripapillary ring scans without pupillary dilation, as previously described.[2] Retinal layer segmentation and thickness measurements were obtained using the device's (Heidelberg Spectralis OCT device (Software version 1.9.10.0); Heidelberg Engineering, Germany) built-in software, including global (pRNFL-G), temporal (pRNFL-T), and papillomacular bundle (pRNFL-PMB) peripapillary retinal nerve fiber layer thicknesses, as well as measurements of the ganglion cell layer (GCL), inner plexiform layer (IPL), and inner nuclear layer (INL). The mean thickness of the macular GCL, IPL, and INL was computed by averaging the parafoveal quadrants of the device-defined fovea-centered Early Treatment Diabetic Retinopathy Study (ETDRS)-style grid with diameters of 1.00, 2.22, and 3.45 mm, and the central foveal subfield was not included.[2] GCIPL thickness was defined as the summation of the GCL and the IPL. All scans included for further analysis were required to meet the OSCAR-IB quality consensus criteria.[3,4] Scans were reviewed and, if necessary, segmentation boundaries were adjusted manually as determined by the consensus of an experienced operator (D.A.), and a senior physician (V. K.). Data from scans were reported in accordance with the Advised Protocol for OCT Study Terminology and Elements (APOSTEL) recommendations.[5,6] Scans not meeting OSCAR-IB quality consensus

criteria or displaying visually apparent image quality issues were excluded from further analysis.[3,4]

Baseline comparisons in the aHSCT cohort

Baseline clinical characteristics and demographics across the three disease subtypes (RRMS, SPMS, PPMS) within the aHSCT cohort were summarized using descriptive statistics, including mean, standard deviation (SD), minimum, and maximum values. Baseline EDSS scores were defined as the last EDSS measurement obtained prior to aHSCT for each participant and summarized using the median, interquartile range (IQR), minimum, and maximum, with quartiles computed from the empirical distribution without interpolation. NEDA-3 maintenance at the last follow-up was defined as the absence of NEDA-3 loss from baseline until the last available clinical assessment.

For continuous variables (age at transplantation, interval between disease onset and aHSCT, and baseline retinal layer thicknesses) normality was assessed using the Shapiro-Wilk test. Normally distributed variables were evaluated for homogeneity of variances using Levene's test. When both normality and homoscedasticity were satisfied, an analysis of variance (ANOVA) was performed; otherwise, Welch's ANOVA was applied. For non-normally distributed variables, the Kruskal-Wallis test was utilized. Significant differences were followed by post hoc pairwise comparisons using Tukey's Honest Significant Difference (HSD) test for ANOVA or Dunn's test with Benjamini-Hochberg (BH) correction for Kruskal-Wallis.

Baseline EDSS was compared across groups using a Kruskal–Wallis test, followed by post hoc pairwise Dunn's tests with BH adjustment. Categorical variables (including sex distribution, number of subjects with NEDA-3 maintenance at last follow-up, and history of ON) were compared across subtypes using the Chi-square test with Yates' continuity correction. When the assumptions for the Chi-square test were violated, Fisher's exact test was employed.

Baseline comparisons of people with RRMS in the aHSCT and control cohort

The highest EDSS score for each participant was defined as the maximum EDSS value ever recorded and reported as the median, empirical IQR, minimum, and maximum. Baseline EDSS in the control cohort was defined as the first available EDSS assessement during the observational period. For continuous variables (age at disease onset, maximum recorded EDSS score, age at first OCT scan, disease duration at first OCT, duration of OCT follow-up, and baseline retinal layer thickness) normality was assessed using the Shapiro-Wilk test. Normally distributed variables were evaluated for homogeneity of variances using Levene's test. If variances were equal, an unpaired Student's t-test was performed; otherwise, Welch's t-test was applied. For non-normally distributed variables and maximum EDSS scores, the Wilcoxon rank-sum test (two-sided) was utilized.

Categorical variables (including sex distribution and the number of eyes with a history of ON) were compared between cohorts using the Chi-square test with Yates' continuity correction. In instances where Chi-square assumptions were not met, Fisher's exact test was applied as an alternative.

Retinal atrophy rates after aHSCT across disease subtypes

Retinal layer thickness changes over time following aHSCT were analysed using LMMs with REML estimation. Separate models were developed for each retinal layer of interest (pRNFL-G, pRNFL-T, pRNFL-PMB, GCIPL, and INL). Fixed effects included days after transplantation, MS subtype (PPMS, SPMS, RRMS), sex, age at transplantation, and an

interaction term between days after transplantation and subtype. A random intercept for each individual was included to account for repeated measurements and inter-eye correlations[7]. The interaction terms were used to estimate atrophy rates by subtype for each retinal layer. Differences in atrophy rates among subtypes were evaluated using two-sided LRT, comparing full models to reduced models, excluding subtypes as a fixed effect. To assess the impact of potential confounders of retinal layer thickness (sex, age at transplantation, time from first manifestations to transplantation, and history of ON) additional models included these variables as fixed effects. LRTs were performed by comparing full models to reduced models, excluding each confounder individually.

Atrophy rates before and after transplantation

To compare changes in retinal atrophy rates prior to and following aHSCT within the transplant cohort, LMMs, fitted via REML estimations, were applied. Longitudinal retinal thickness values (pRNFL-G, pRNFL-T, pRNFL-PMB, GCIPL, and INL) served as the dependent variables. Fixed effects included sex, age at transplantation, days after transplantation, transplant status (pre- or post-transplantation), and an interaction term between days after transplantation and transplant status. Random intercepts for each participant were utilized to account for repeated measurements and within-subject inter-eye correlations. Days since transplantation were recorded as negative values prior to aHSCT and positive values following aHSCT. Transplant status was recorded as a binary factor with two levels, namely pre- and post-transplantation. Models did not account for a history of ON, as both pre- and post-transplantation data stemmed from the same individuals, and models were fit with random intercepts. Model outputs were reported as µm/year to determine absolute estimates of atrophy. Hypothesis testing was conducted by comparing the full models to reduced models lacking the interaction term between days after transplantation and transplant status, both fitted via ML estimations, by performing an LRT.
To evaluate whether heterogeneity in pre-aHSCT scan timing influenced estimated pre/post atrophy-rate differences, additional LMMs were fitted with pre-aHSCT timing descriptors included as covariates. Timing descriptors were defined at the eye level and included the interval between aHSCT and the closest pre-aHSCT OCT scan, the interval between aHSCT and the earliest pre-aHSCT OCT scan, and the number of available pre-aHSCT OCT scans. For each timing-adjusted model, the post-minus-pre slope difference was extracted and compared with the unadjusted model. Statistical significance was assessed using LRTs comparing the full model including the days-after-aHSCT × transplant-status interaction term with a reduced model lacking said interaction term.
To examine the influence of aberrant measurements on model estimates, an observation-level outlier sensitivity analysis was performed. For each retinal layer, conditional residuals were extracted from the corresponding RRMS pre/post LMM and standardized by the residual standard deviation. Observations with an absolute standardized residual greater than 2 were classified as outliers. The primary RRMS pre/post models were then refitted after excluding these observations, and the resulting post-minus-pre slope estimates and LRT-derived p-values were compared with the full-data estimates.

Atrophy rates in transplanted and reference people with RRMS

To investigate if atrophy rates in transplanted individuals differ relative to an observational control cohort, LMMs fitted with REML estimations were applied. Longitudinal retinal thickness values represented the dependent variables, and models were fitted with random intercepts for each participant. Fixed effects included years since disease onset, sex, age at onset, cohort (aHSCT or Control), the highest ever recorded EDSS score, baseline retinal layer

thickness, and an interaction term between years since disease onset and cohort (aHSCT or Control). Years since disease onset served as a fixed effect to determine changes in retinal thickness over time, implicitly accounting for disease duration. For these between-cohort analyses, baseline was defined as the first available post-aHSCT OCT scan in the transplanted cohort and the first available OCT scan in the observational control cohort. In eyes with an episode of ON during the data collection period, all images obtained up to six months before ON onset or after the ON episode were removed from further analysis.[8] Hypothesis testing was conducted by comparing the full LMMs, fitted with ML estimations, to reduced models lacking the interaction term between years since disease onset and cohort (aHSCT or Control) by performing an LRT.

Sensitivity to baseline retinal thickness imbalance between cohorts was evaluated by comparing three model specifications. We fitted an unadjusted model without baseline retinal thickness as a covariate, a baseline-adjusted model including baseline retinal thickness as a fixed effect, and an interaction-adjusted model additionally including a YSO × baseline retinal thickness interaction. Model fit was compared across specifications using LRTs and AIC. For each specification, estimated between-cohort atrophy-rate differences and corresponding LRT-derived p-values were reported.

Robustness to differential follow-up duration was evaluated by restricting the control cohort to progressively narrower central windows of the aHSCT YSO distribution, while keeping the aHSCT cohort unchanged. A central X% window was defined as the interval containing the central X% of aHSCT observations after excluding equal proportions from both tails of the YSO distribution. Analyses were repeated for the central 100%, 95%, 90%, 85%, 80%, 75%, and 70% windows. For each restricted control cohort, baseline-adjusted LMMs were refitted, and between-cohort slope differences and LRT-derived p-values were reported.

Association of baseline OCT measurements with neurological disability after transplantation

To evaluate whether baseline average (avg.) retinal layer thicknesses predicted longitudinal neurological disability following aHSCT, we employed cumulative link mixed models (CLMMs) fitted with the Laplace approximation. OCT images from the last imaging session before transplantation were retrieved, and the extracted thickness values were averaged across both eyes for all layers. In case a history of ON was present in an eye or a scan did not meet image quality criteria, only thickness values from the contralateral eye of a participant were utilized. These extracted averaged thickness values for each retinal layer were referred to as baseline average (avg.) thickness. Only EDSS values recorded after transplantation were considered in the models. Each CLMM was fitted separately for relapsing-remitting MS (RRMS) and progressive MS subgroups. The models included age at transplantation, pre-aHSCT EDSS, baseline avg. thickness of the layer of interest, years since transplantation, an interaction term between time after aHSCT and the respective retinal layer thickness variable, along with random intercepts for each individual. To assess the statistical significance of the interaction terms, full models (including the interaction term) were compared to reduced models (excluding the interaction term) using LRTs. Effect sizes were converted from log-odds scale to interpretable odds ratios (ORs) by exponentiating the estimated coefficients. To complement this analysis, we grouped individuals based on whether their baseline retinal layer thickness was above or below the group-specific mean. Separate CLMMs were then fit using group (Above vs. Below), time, and their interaction as predictors. Odds ratios for the interaction term represent the change in odds of EDSS progression per year for individuals with above-mean vs. below-mean baseline thickness. For visualization, model-predicted EDSS trajectories over time were computed, estimating category-specific predicted probabilities from the fitted CLMM.

These probabilities were then used to derive numerical expected EDSS scores by calculating the probability-weighted sum across EDSS categories at each time point. These continuous expected EDSS values were plotted and overlaid with actual individual trajectories for EDSS measurements, providing both model-based and observed perspectives on post-aHSCT disability evolution.
To assess robustness of the CLMM estimates, patient-level bootstrap confidence intervals were derived for the interaction effects using 500 bootstrap resamples with a fixed random seed. Participants were resampled with replacement, and the same CLMM specification was refitted in each bootstrap sample. Leave-one-patient-out (LOPO) analyses were additionally performed by iteratively refitting each CLMM after excluding one participant at a time. For each model, the bootstrap median odds ratio, 95% bootstrap confidence interval, LOPO odds-ratio range, presence of direction reversals, and number of statistically significant refits were reported. The maximum absolute optimizer gradient was reported as a numerical model diagnostic value.

Supplementary References

1 Ruder, J. *et al.* NK Cells and Innate-Like T Cells After Autologous Hematopoietic Stem Cell Transplantation in Multiple Sclerosis. *Front Immunol* **12**, 794077 (2021). https://doi.org/10.3389/fimmu.2021.794077

2 Wicki, C. A., Manogaran, P., Simic, T., Hanson, J. V. M. & Schippling, S. Bilateral retinal pathology following a first-ever clinical episode of autoimmune optic neuritis. *Neurol Neuroimmunol Neuroinflamm* **7** (2020). https://doi.org/10.1212/NXI.0000000000000671

3 Schippling, S. *et al.* Quality control for retinal OCT in multiple sclerosis: validation of the OSCAR-IB criteria. *Multiple sclerosis* **21**, 163-170 (2015). https://doi.org/10.1177/1352458514538110

4 Tewarie, P. *et al.* The OSCAR-IB consensus criteria for retinal OCT quality assessment. *PloS one* **7**, e34823 (2012). https://doi.org/10.1371/journal.pone.0034823

5 Aytulun, A. *et al.* APOSTEL 2.0 Recommendations for Reporting Quantitative Optical Coherence Tomography Studies. *Neurology* **97**, 68-79 (2021). https://doi.org/10.1212/WNL.0000000000012125

6 Cruz-Herranz, A. *et al.* The APOSTEL recommendations for reporting quantitative optical coherence tomography studies. *Neurology* **86**, 2303-2309 (2016). https://doi.org/10.1212/WNL.0000000000002774

7 Button, J. *et al.* Disease-modifying therapies modulate retinal atrophy in multiple sclerosis: A retrospective study. *Neurology* **88**, 525-532 (2017). https://doi.org/10.1212/WNL.0000000000003582

8 Bsteh, G. *et al.* Validation of inter-eye difference thresholds in optical coherence tomography for identification of optic neuritis in multiple sclerosis. *Mult Scler Relat Disord* **45**, 102403 (2020). https://doi.org/10.1016/j.msard.2020.102403

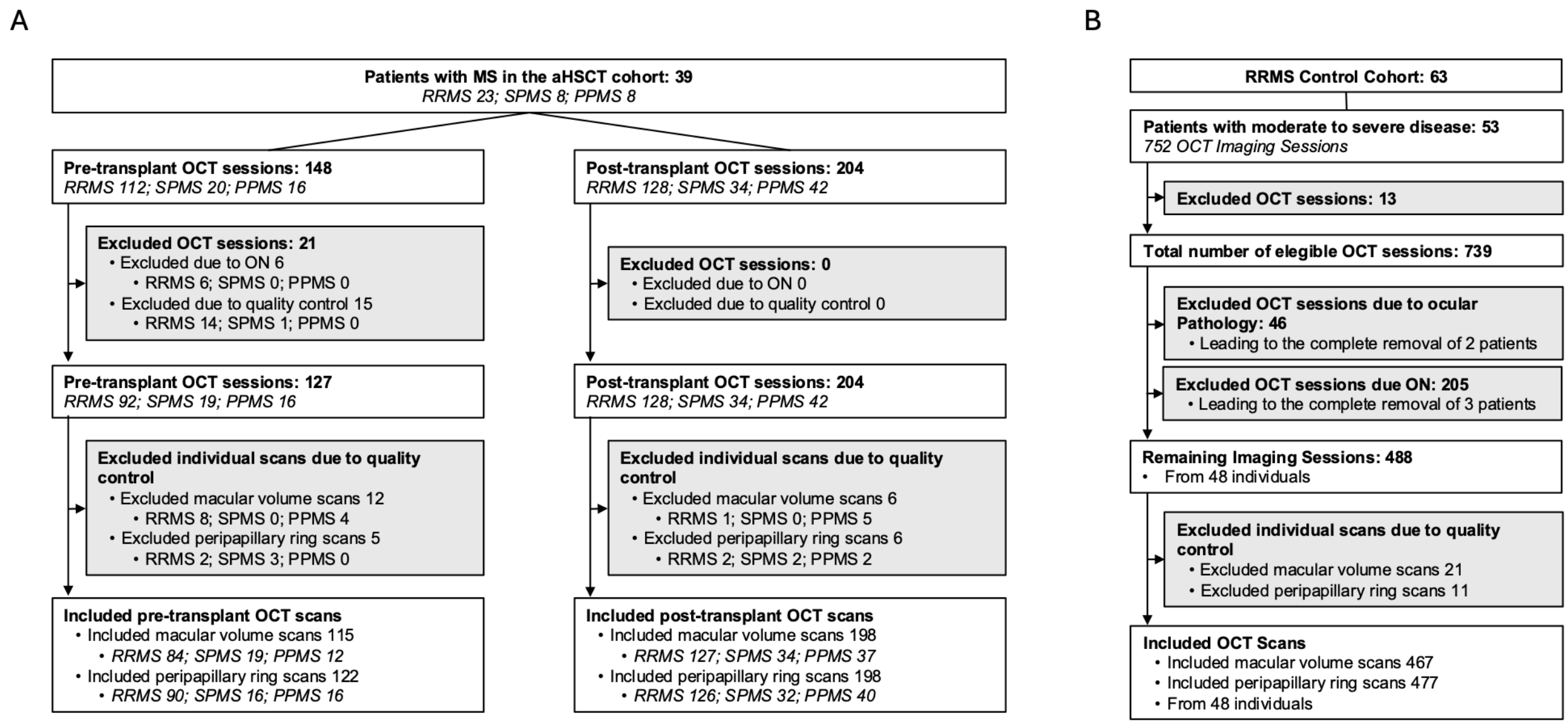

Supplementary Figure 1 Flowchart of study design for the (A) aHSCT cohort and (B) control cohort.
aHSCT autologous hematopoietic stem cell transplantation, *MS* multiple sclerosis, *OCT* Optical Coherence Tomography, *ON* optic neuritis, *PPMS* primary progressive multiple sclerosis, *RRMS* relapsing-remitting multiple sclerosis, *SPMS* secondary progressive multiple sclerosis.

Alt text: Two-panel flow diagram depicting the selection of optical coherence tomography scans for the autologous hematopoietic stem cell transplantation and control cohorts included in the final analysis. Exclusion criteria were optic neuritis episodes, a history of ocular pathology, and insufficient image quality.

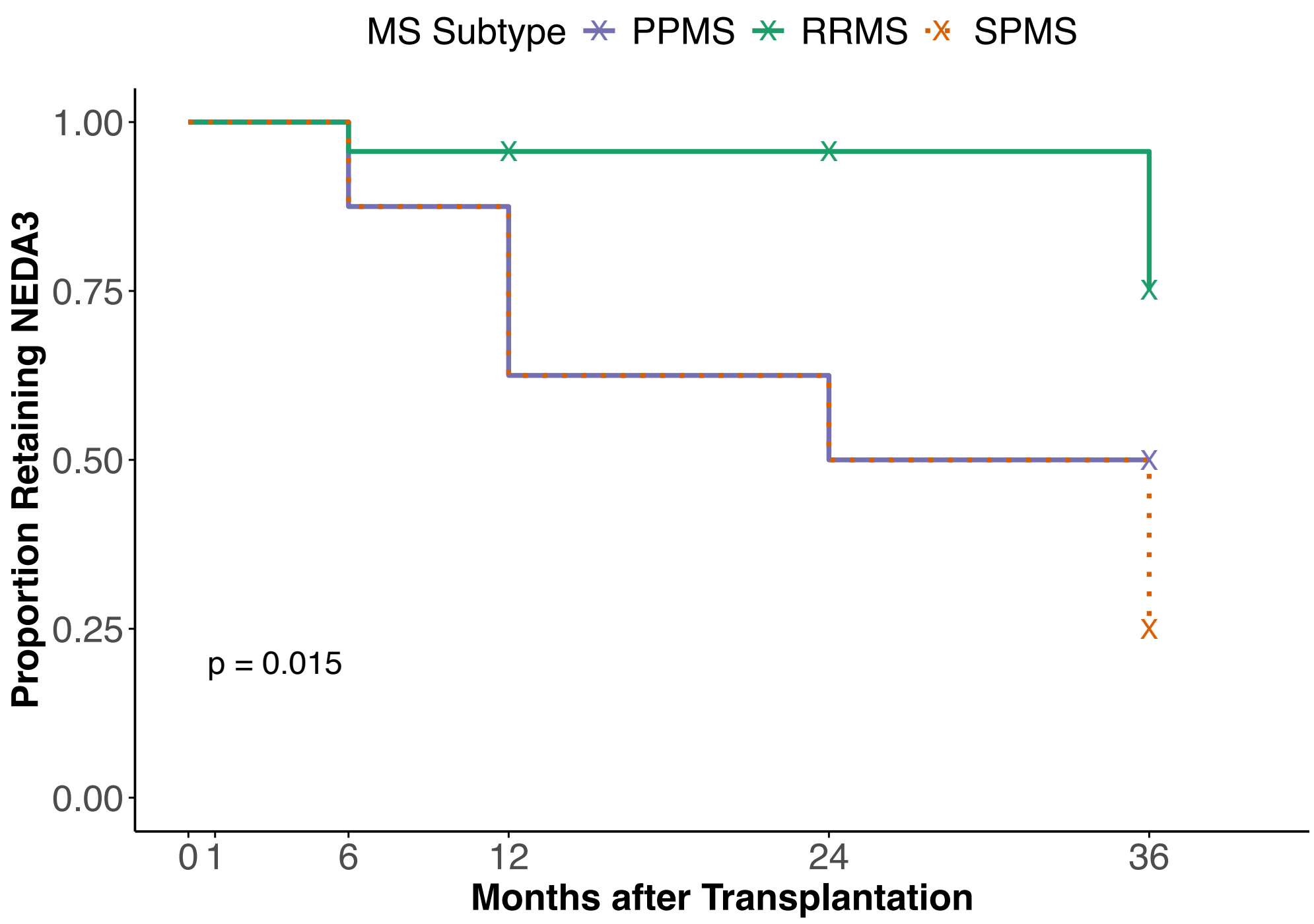

Supplementary Figure 2 No Evidence of Disease Activity (NEDA-3) maintenance after autologous hematopoietic stem cell transplantation by disease subtype. Kaplan-Meier survival curves showing the proportion of individuals retaining NEDA-3 over time across multiple sclerosis (MS) subtypes: PPMS (Primary Progressive MS, violet, solid line), RRMS (Relapsing-Remitting MS, green, solid line), and SPMS (Secondary Progressive MS, orange, dotted line). Survival probabilities are displayed with censoring indicated by "X" markers. The x-axis represents months after transplantation, and the y-axis indicates the proportion retaining NEDA-3. The p-value from the overall log-rank test is included to show statistically significant differences across the subtypes. Differences in NEDA-3 maintenance across subtypes were assessed using a global log-rank test, which showed a statistically significant difference between Kaplan–Meier curves ($\chi^2(2) = 8.41$, $p = 0.015$).

Alt-text: Kaplan–Meier curves showing NEDA-3 retention up to 36 months after autologous hematopoietic stem cell transplantation by multiple sclerosis subtype. Relapsing-remitting multiple sclerosis shows higher NEDA-3 maintenance than progressive subtypes.

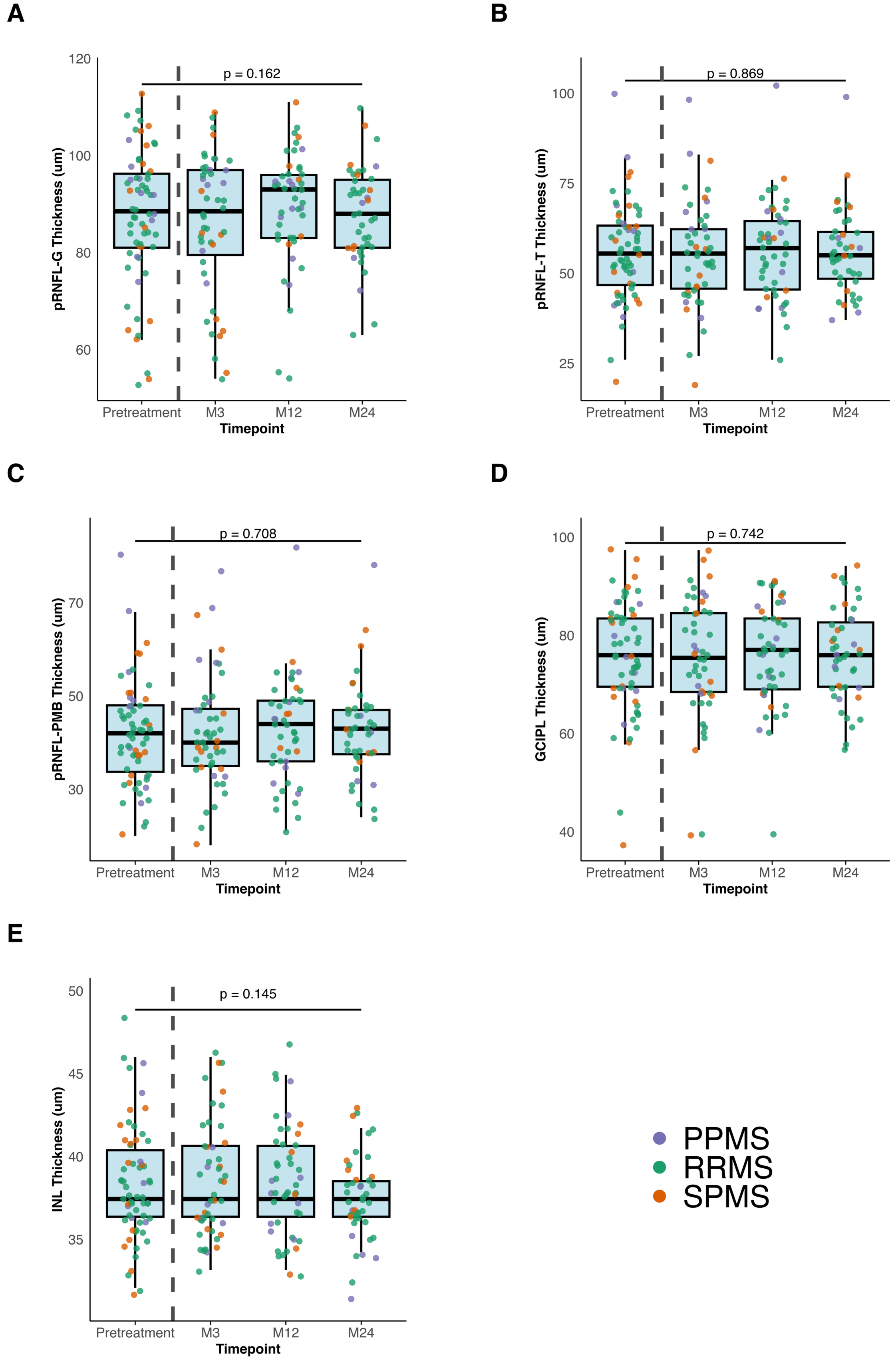

Supplementary Figure 3 Retinal layer thickness following transplantation. Boxplots of retinal layer thickness following transplantation, showing the distribution of measurements (in micrometers) for (**A**) global peripapillary retinal nerve fiber layer (pRNFL-G), (**B**) temporal

peripapillary retinal nerve fiber layer (pRNFL-T), (**C**) papillomacular bundle of the peripapillary retinal nerve fiber layer (pRNFL-PMB), (**D**) ganglion cell-inner plexiform layer (GCIPL), and (**E**) inner nuclear layer (INL). Boxplots represent the median (horizontal line) and interquartile range (IQR; box boundaries), with whiskers extending to the smallest and largest values within 1.5 times the IQR from the lower and upper quartiles, respectively. Overlaid scatter points represent individual measurements, color-coded by multiple sclerosis subtype (green: relapsing-remitting multiple sclerosis (RRMS), orange: secondary progressive multiple sclerosis (SPMS), purple: primary progressive multiple sclerosis (PPMS)), with all points displayed. Horizontal black bars denote hypothesis testing results from likelihood-ratio tests comparing linear mixed-effects models with and without timepoint as a fixed effect, with p-values annotated. Retinal thickness changes are shown at Pretreatment ($n = 64$ eyes from 32 individuals), 3 months (M3; $n = 48$ eyes from 24 individuals), 12 months (M12; $n = 47$ eyes from 24 individuals), and 24 months (M24; $n = 44$ eyes from 22 individuals). Observations at M36 were excluded due to limited data. Test statistics were: pRNFL-G, $\chi^2(3) = 5.13$, $p = 0.162$; pRNFL-T, $\chi^2(3) = 0.72$, $p = 0.869$; pRNFL-PMB, $\chi^2(3) = 1.39$, $p = 0.708$; GCIPL, $\chi^2(3) = 1.25$, $p = 0.742$; and INL, $\chi^2(3) = 5.40$, $p = 0.145$.

Alt text: Box-and-whisker plots showing retinal layer thickness before and 3, 12, and 24 months after autologous hematopoietic stem cell transplantation. Retinal thickness remains stable over time across all multiple sclerosis subtypes.

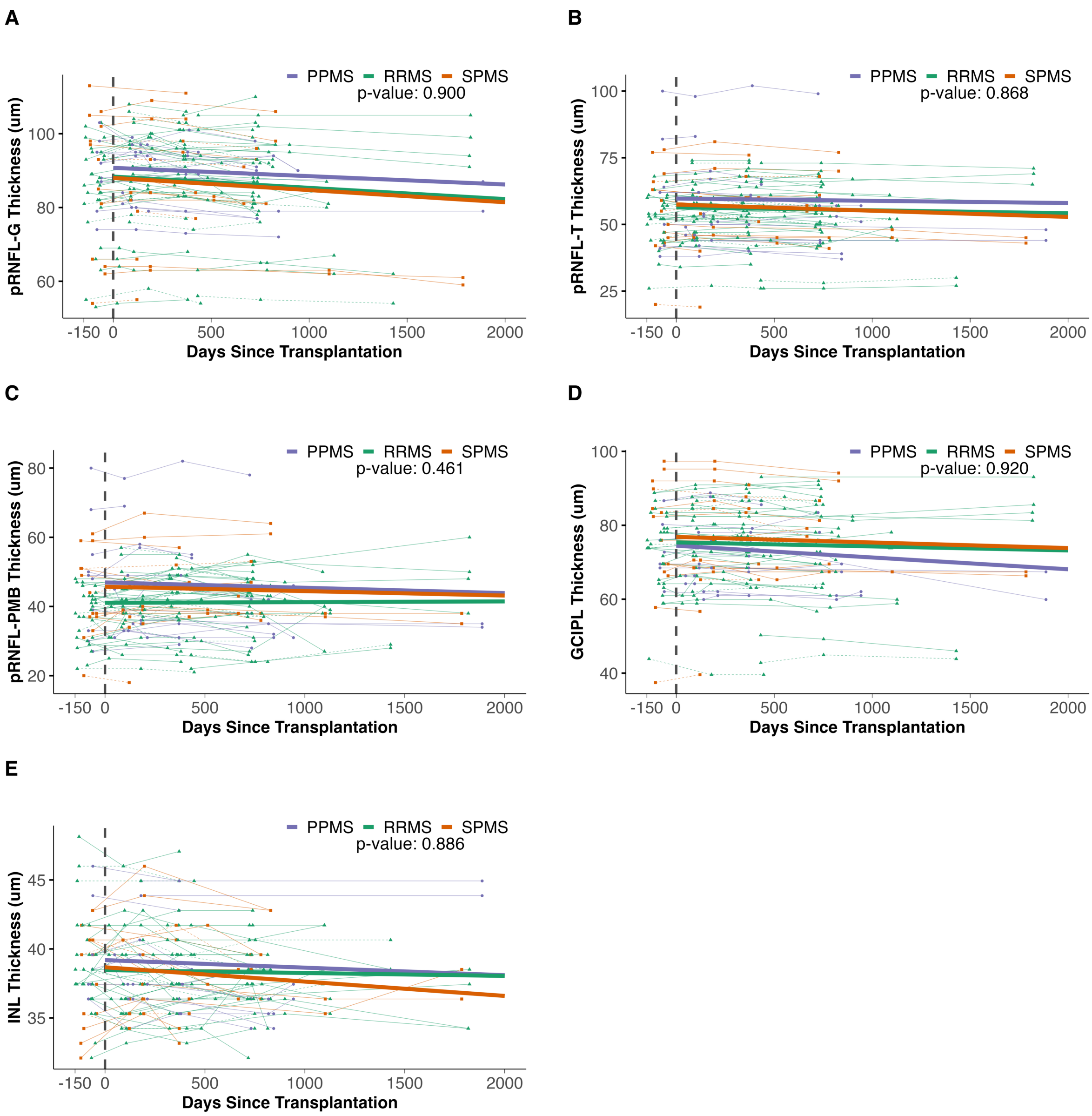

Supplementary Figure 4 Retinal thickness measurements after transplantation by multiple sclerosis (MS) subtype. Analyses were carried out with linear mixed-effects models (LMM). Each panel represents measurements for a different retinal layer: (**A**) global peripapillary retinal nerve fiber layer (pRNFL-G), (**B**) temporal peripapillary retinal nerve fiber layer (pRNFL-T), (**C**) papillomacular bundle of the peripapillary retinal nerve fiber layer (pRNFL-PMB), (**D**) ganglion cell-inner plexiform layer (GCIPL), and (**E**) inner nuclear layer (INL). The panels show the relationship between days post-transplantation and retinal layer thickness, with multiple sclerosis (MS) subtypes differentiated by color and history of optic neuritis (ON) by line shape. Relapsing-remitting MS (RRMS) (n = 23) in green, secondary progressive MS (SPMS) (n = 8) in orange, and primary progressive MS (PPMS) (n = 8) in violet.

Each point represents one eye-level retinal thickness measurement. Fine lines connect repeated measurements from the same eye over time. Bold lines represent model-estimated retinal atrophy rates, computed individually for each subtype. Vertical dashed lines indicate the day of autologous hematopoietic stem cell transplantation (aHSCT), with intercepts between bold lines and the vertical dashed line representing the model-estimated intercept at transplantation. For

categorical predictors, the mode was used in model estimates, while for continuous predictors, the cohort median was applied. The p-values shown in each panel were calculated using likelihood-ratio tests comparing full and reduced LMMs. Test statistics were: pRNFL-G, $\chi^2(2) = 0.21$, $p = 0.900$; pRNFL-T, $\chi^2(2) = 0.28$, $p = 0.868$; pRNFL-PMB, $\chi^2(2) = 1.55$, $p = 0.461$; GCIPL, $\chi^2(2) = 0.17$, $p = 0.920$; and INL, $\chi^2(2) = 0.24$, $p = 0.886$.

Alt text: Longitudinal retinal thickness trajectories after autologous hematopoietic stem cell transplantation by multiple sclerosis subtype. Individual measurements and model-estimated trends show no significant differences in retinal atrophy rates between disease subtypes.

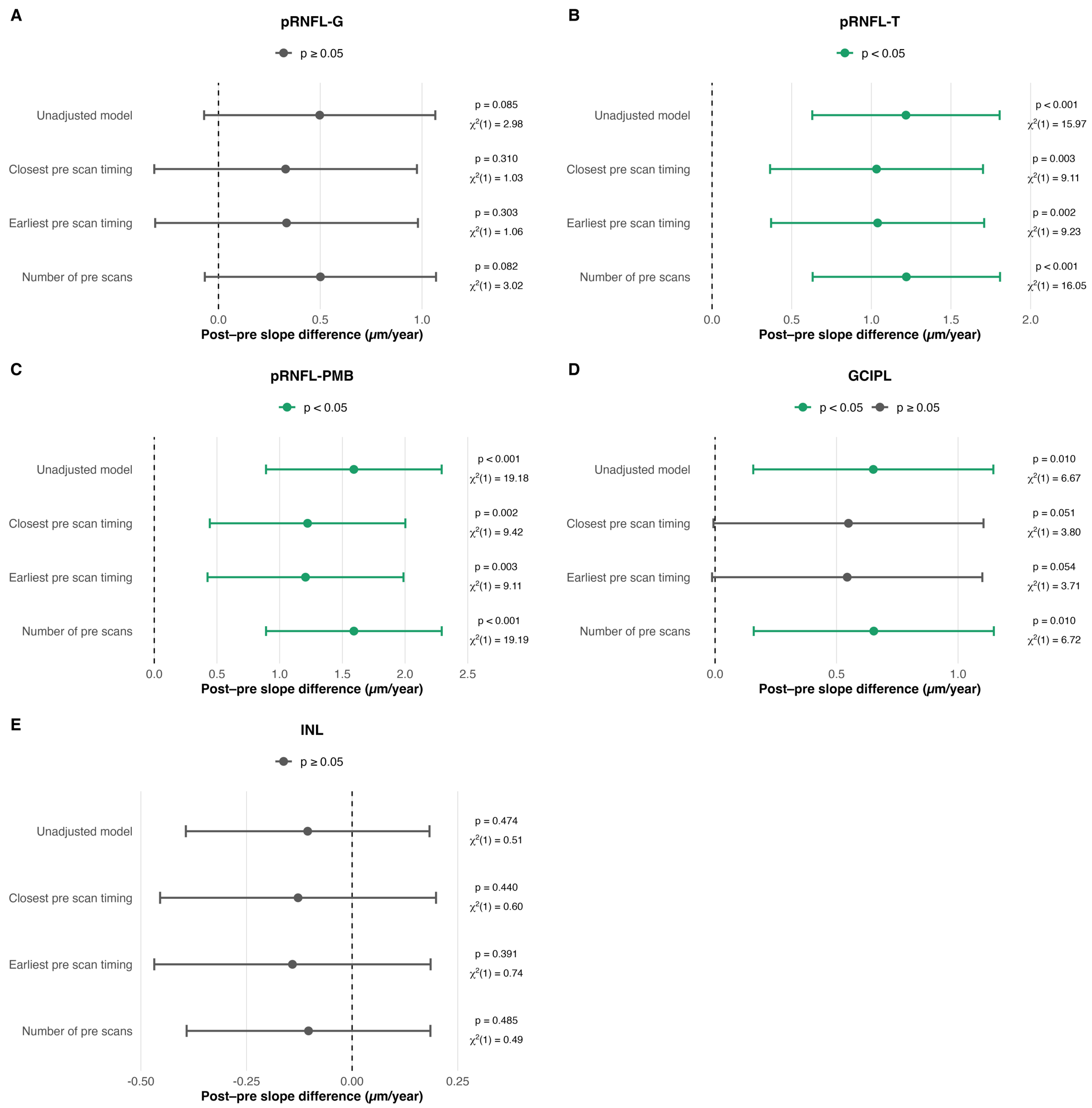

Supplementary Figure 5 Effect of pre-autologous hematopoietic stem cell transplantation (aHSCT) timing covariates on estimated pre/post retinal atrophy differences in relapsing-remitting multiple sclerosis (RRMS). Forest plots showing the estimated post-minus-pre aHSCT difference in yearly atrophy rate for the base statistical model and in models additionally adjusted for pre-aHSCT timing covariates for (**A**) global peripapillary retinal nerve fiber layer (pRNFL-G), (**B**) temporal peripapillary retinal nerve fiber layer (pRNFL-T), (**C**) papillomacular bundle of the peripapillary retinal nerve fiber layer (pRNFL-PMB), (**D**) ganglion cell-inner plexiform layer (GCIPL), and (**E**) inner nuclear layer (INL) for RRMS participants (n=23). Timing covariates shown are the interval from transplantation to the closest pre-aHSCT scan, the interval to the earliest pre-aHSCT scan, and the number of pre-aHSCT scans. Points represent model estimates, and horizontal bars indicate 95% confidence intervals. The χ^2 test-statistics and p-values shown were calculated using likelihood-ratio tests for the post/pre slope difference in the corresponding linear mixed-effects models. Positive values indicate less thinning after aHSCT than before aHSCT.

Alt text: Forest plots comparing estimated pre- versus post-transplant retinal atrophy rate differences across five retinal layers using alternative statistical models. Results remain consistent across sensitivity analyses, with significant attenuation of atrophy in selected retinal layers.

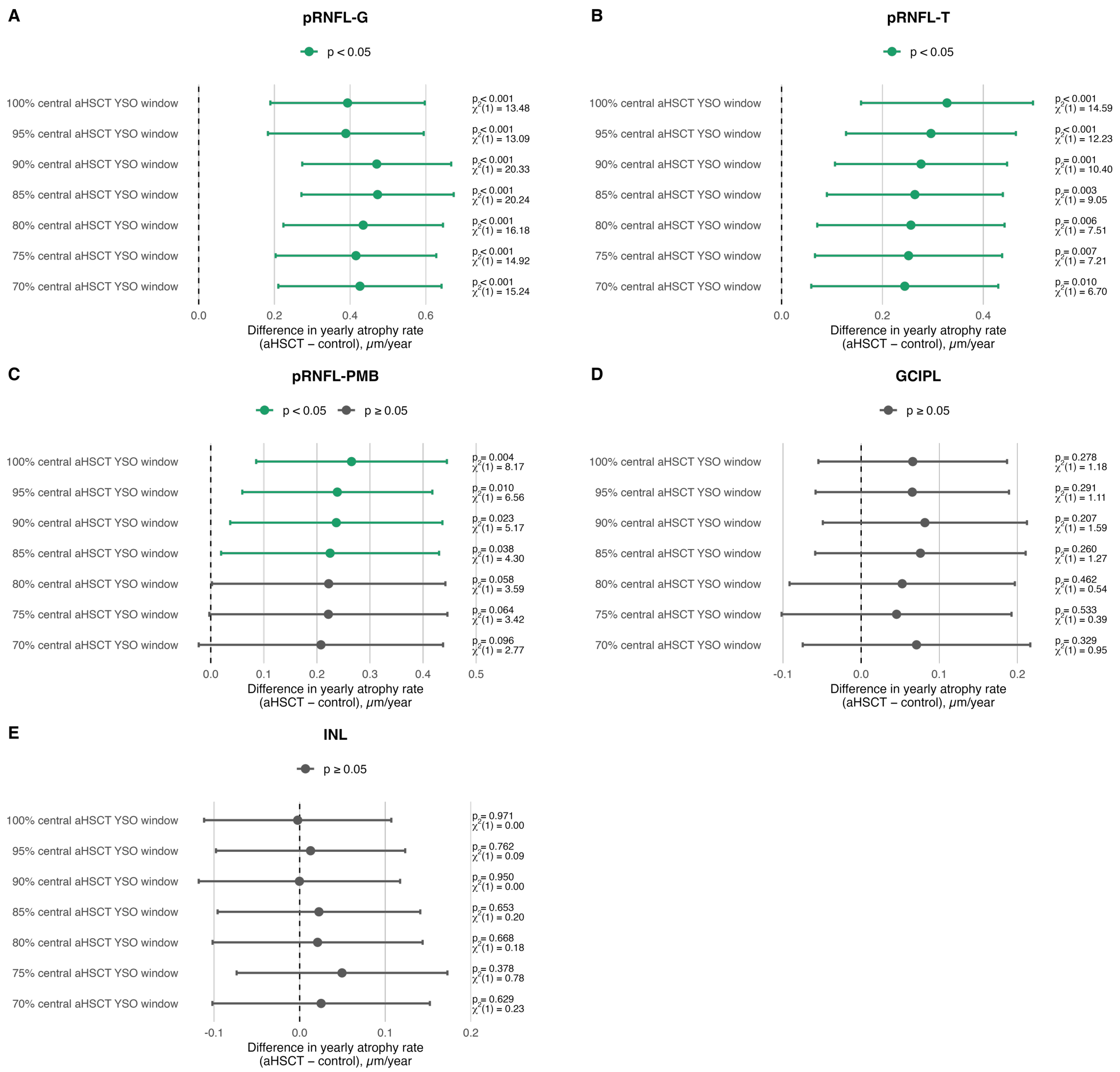

Supplementary Figure 6 Sensitivity analysis for differential follow-up duration between the control and autologous hematopoietic stem cell transplantation (aHSCT) cohort. Forest plots show the estimated between-cohort difference in yearly retinal atrophy rate (aHSCT − control) for each retinal layer after progressively restricting the control cohort to the central 100%, 95%, 90%, 85%, 80%, 75%, and 70% of the years-since-onset (YSO) distribution observed in the aHSCT cohort, while keeping the aHSCT cohort unchanged. Panels represent the five different retinal layers analyzed with (**A**) global peripapillary retinal nerve fiber layer (pRNFL-G), (**B**) temporal peripapillary retinal nerve fiber layer (pRNFL-T), (**C**) papillomacular bundle of the peripapillary retinal nerve fiber layer (pRNFL-PMB), (**D**) ganglion cell-inner plexiform layer (GCIPL), and (**E**) inner nuclear layer (INL) for RRMS participants (n=23). Points represent model estimates from baseline-adjusted linear mixed-effects models, horizontal bars indicate 95% confidence intervals, and point color denotes statistical significance of the cohort-by-YSO interaction. The p-values and χ^2 test-statistics were calculated using likelihood-ratio tests for the cohort-by-YSO interaction in the corresponding models, and point color indicates statistical significance. Positive values indicate less thinning in the aHSCT cohort than in controls. Demographic information corresponding to the control cohorts derived from limiting to the defined YSO window are depicted in Supplementary Table 11.

Alt text: Forest plots showing sensitivity analyses comparing retinal atrophy rates between the autologous hematopoietic stem cell transplantation and control cohorts for different follow-up intervals. Findings remain consistent across progressively restricted follow-up durations, supporting the robustness of the results.

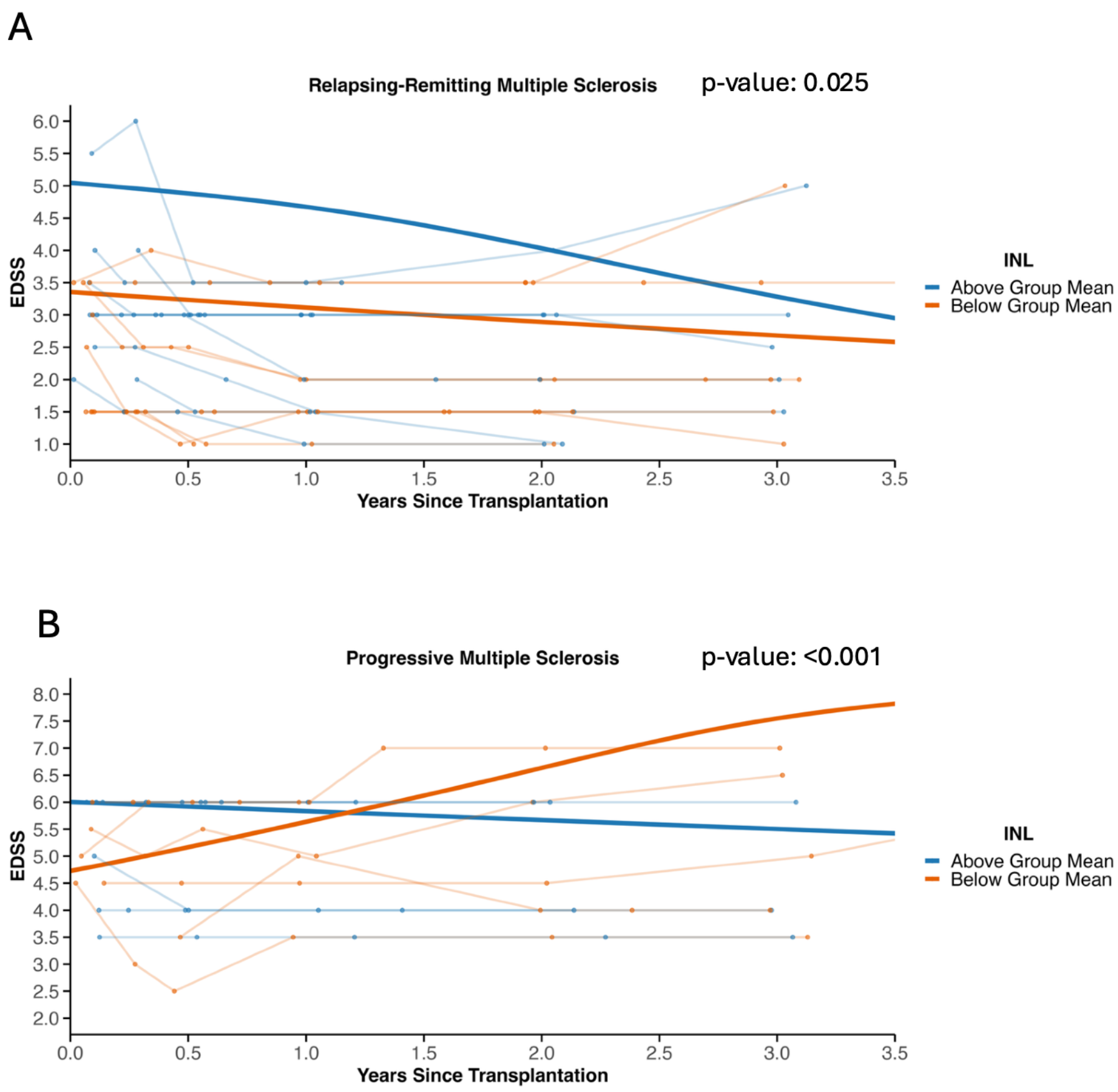

Supplementary Figure 7 Longitudinal Expanded Disability Status Scale (EDSS) trajectories following autologous hematopoietic stem cell transplantation (aHSCT) by baseline inner nuclear layer (INL). The figure shows the expected and observed EDSS trajectories following aHSCT, stratified by baseline INL thickness relative to the group mean. Results are shown separately for (**A**) relapsing-remitting multiple sclerosis (RRMS) and (**B**) progressive multiple sclerosis (MS). INL thickness was measured before aHSCT (mean = 37.95 µm in RRMS; 38.48 µm in progressive MS). Participants were stratified into "Above" and "Below" groups based on their subtype-specific mean INL (RRMS: $n = 10$ above, 9 below; progressive MS: $n = 6$ above, 6 below). Each point represents one observed EDSS score from one post-aHSCT follow-up visit for one participant. Bold lines represent model-derived expected EDSS trajectories, with blue indicating values above and orange below the group mean. Fine lines show observed individual EDSS trajectories using the same color scheme. Expected values were estimated using cumulative link mixed models (CLMMs) including an interaction between time since aHSCT and INL group, adjusted for subject-level random effects. Numerical expected EDSS scores were calculated as the probability-weighted average across all EDSS categories at each timepoint, based on the model-predicted probability distribution. Hypothesis testing was done using likelihood-ratio tests comparing CLMMs with and without the INL group × time-since-aHSCT interaction. Test statistics were: RRMS, $\chi^2(1)= 5.02$; progressive MS, $\chi^2(1)= 11.43$.

Alt text: Scatter plots showing the association between inner nuclear layer thickness before treatment and disability outcomes after autologous hematopoietic stem cell transplantation. Higher baseline inner nuclear layer thickness predicts less disability worsening in relapsing-remitting and progressive multiple sclerosis.

Supplementary Table 1 Summary of clinical and imaging data in the aHSCT cohort by disease subtype.

	**Overall aHSCT (n = 39)**	**RRMS aHSCT (n = 23)**	**SPMS aHSCT (n = 8)**	**PPMS aHSCT (n = 8)**
**Number of EDSS assessments**				
Total	420	245	79	96
Before Transplantation	202	112	42	48
After Transplantation	218	133	37	48
Follow-Up (Max. days; Mean, SD)	989; 435<br>191-1913	973; 452<br>191-1913	919; 460<br>354-1785	1104; 392<br>442-1888
**Number Imaging Sessions**				
Total	352	240	54	58
Included	331	220	53	58
Before Transplantation	127	92	19	16
After Transplantation	204	128	34	42
Excluded due to Image Quality Issues (All Pre)	15	14	1	0
Excluded due to ON (All Pre)	6	6	0	0
Follow-Up after aHSCT (Max. Days; Mean, SD)<br>Min, Max	805; 439<br>84-1887	827; 425<br>84-1824	713; 496<br>121-1785	834; 467<br>371-1887
**Macular Volume Scans**				
Total Included	313	211	53	49
Before Transplantation	115	84	19	12
After Transplantation	198	127	34	37
Excluded due to Image Quality Issues	18	9	0	9
**Peripapillary Ring Scans**				
Total Included	320	216	48	56
Before Transplantation	122	90	16	16
After Transplantation	198	126	32	40
Excluded due to Image Quality Issues	11	4	5	2
**Subjects without**				
Pre-Transplant OCT Measurements	7	3	1	3
Post-Transplant OCT Measurements	0	0	0	0

Overview of clinical and imaging assessments within the autologous hematopoietic stem cell transplantation (aHSCT) cohort, categorized by disease subtype (relapsing-remitting multiple sclerosis (RRMS), secondary progressive multiple sclerosis (SPMS), and primary progressive multiple sclerosis (PPMS)). The total number of expanded disability status scale (EDSS) assessments is reported for both pre- and post-transplantation periods, accompanied by follow-up duration in days (mean ± standard deviation (SD) and range) for each subtype. Additionally, the table outlines the total number of imaging sessions conducted, specifying the total number of sessions included in the analysis and the number excluded due to insufficient scan quality or history of optic neuritis (ON). Follow-up durations are also presented as mean ± SD and range values. The table further details the total count of macular volume scans and peripapillary ring scans used in the analysis, with pre- and post-transplantation counts delineated alongside the number of individual scans excluded due to image quality issues. Lastly, the table specifies the count of participants lacking pre- or post-transplant optical coherence tomography (OCT) measurements in each subtype group, highlighting instances where retinal imaging data were unavailable.

Supplementary Table 2 Demographics and clinical characteristics of the aHSCT cohort

	All MS	RRMS	SPMS	PPMS	*p*-value
**Subjects, *n* (%)**	39	23 (59)	8 (20.5)	8 (20.5)	
**Female, *n* (%)**	23 (59)	15 (65.2)	5 (62.5)	3 (37.5)	0.439[a]
**Age at aHSCT, y**					**<0.001**[b, i]
mean (SD)	40.3 (8.11)	36.1 (5.7)	47.2 (5.9)	45.3 (8.8)	
min; max	25.5; 54.3	25.5; 47.5	36.6; 54.3	26; 53.6	
**Duration between disease onset[x] and aHSCT, y**					0.145[c]
mean (SD)	9.4 (5.6)	8.4 (6.2)	12.8 (5.5)	8.6 (3.7)	
min; max	0.6; 21.2	0.6; 21.2	6.9; 20.5	3.4; 20.5	
**EDSS before aHSCT**					**<0.001**[d, i]
median (empirical IQR)	4 (3 – 5)	3 (2 – 4)	4.5 (4 – 5)	6 (4 – 6)	
min; max	1.5; 6.5	1.5; 5.5	3.5; 6.5	4; 6.5	
**Subjects with NEDA-3 maintenance at last follow-up, *n* (%)**	25 (64.1)	19 (82.6)	2 (25)	4 (50)	**0.009**[e, i]
**Eyes included in analysis, *n***	78	46	16	16	
**Eyes with history of ON[g], *n***	13[h]	9	4	0	0.062[f]

aHSCT autologous hematopoietic stem cell transplantation, *EDSS* expanded disability status scale, *IQR* interquartile range, *MS* multiple sclerosis, *max* maximum, *min* minimum, *n* number, *NEDA-3* No Evidence of Disease Activity, *ON* optic neuritis, *PPMS* primary progressive multiple sclerosis, *RRMS* relapsing-remitting multiple sclerosis, *SD* standard deviation, *SPMS* secondary progressive multiple sclerosis, *y* years.

[a] Fisher's Exact test (two-sided).
[b] ANOVA, F(2, 36)= 12.06, with two-sided Tukey HSD for post hoc pairwise comparisons (RRMS vs. PPMS p= 0.003, q=4.9; RRMS vs. SPMS p <0.001, q=5.98; SPMS vs. PPMS p= 0.827, q=0.83).
[c] Kruskal-Wallis test, $\chi^2(2)$= 3.85.
[d] Kruskal–Wallis test, $\chi^2(2)$= 20.54, with Dunn's test for post hoc pairwise comparisons using BH correction (RRMS vs PPMS p < 0.001, z= 3.76: RRMS vs SPMS p = 0.003, z= 3.09: SPMS vs PPMS p = 0.46, z= 0.74).
[e] Fisher's exact test (two-sided) with BH-adjusted pairwise post hoc comparisons (RRMS vs SPMS p=0.018, RRMS vs PPMS p=0.231, SPMS vs PPMS p=0.608).
[f] Fisher's Exact test (two-sided).
[g] ON before transplantation; no ON occurred after aHSCT.
[h] Only unilateral, no bilateral ON occurred.
[i] bold values represent p-values below the threshold of statistical significance at alpha of 0.05.

x self-reported first manifestation of symptoms suggestive of MS; diagnosis can be later.

Supplementary Table 3 Demographics and clinical characteristics of people with RRMS in observational control and aHSCT cohorts

	RRMS control	RRMS aHSCT	*p*-value
**Subjects, *n* (%)**	48	23	
**Female, *n* (%)**	31 (64.58)	15 (65.2)	1[a]
**Age at disease onset, y**			0.115[b]
mean (SD)	30.8 (7.95)	27.6 (6.6)	
min; max	16.8; 50.2	17.5; 42.1	
**Maximum recorded EDSS, absolute**			
median (empirical IQR)	2 (1 – 2.5)	3 (2 - 4)	**<0.001**[c, i]
min; max	0; 4	1.5; 6	
**Eyes included in analysis, *n***	77	46	
**Eyes with history of ON, *n***	31[h]	9	0.077[d]
**Age at first OCT, y**			0.098[e]
mean (SD)	34.7, 9.6	36.5 (5.8)	
min; max	16.9; 63.9	25.7; 48.0	
**Disease duration at first OCT, y**			**<0.001**[f, i]
mean (SD)	3.9 (6.2)	8.9 (6.2)	
min; max	0.01; 26.4	0.6; 21.4	
**Duration of OCT follow-up, y**			**<0.001**[g, i]
mean (SD)	4.7 (2.3)	2.3 (1.2)	

aHSCT autologous hematopoietic stem cell transplantation, *d* days, *EDSS* expanded disability status scale, *IQR* interquartile range, *max* maximum, *min* minimum, *n* number, *OCT* optical coherence tomography, *ON* optic neuritis, *RRMS* relapsing-remitting multiple sclerosis, *SD* standard deviation, *y* years.

[a] Chi-square test with Yates' continuity correction (two-sided), $\chi^2(1)= 0.00$
[b] Wilcoxon rank sum test (two-sided), W= 681.
[c] Wilcoxon rank sum test (two-sided), W= 235.
[d] Chi-square test with Yates' continuity correction (two-sided), $\chi^2(1)= 3.13$.
[e] Wilcoxon rank sum test (two-sided), W= 417.
[f] Wilcoxon rank sum test (two-sided), W= 232.
[g] Wilcoxon rank sum test (two-sided), W= 902.
[h] 7 individuals with both eyes affected by ON (consecutive ON, not bilateral ON).
[i] bold values represent p-values below the threshold of statistical significance at alpha of 0.05.

Supplementary Table 4 Disease modifying treatment in relapsing-remitting MS control cohort

Subject ID	Substance
**1**	Interferon beta-1b, Glatiramer acetate, Dimethyl fumarate, Fingolimod, Natalizumab
**2**	Interferon beta-1a, Dimethyl fumarate, Rituximab
**10**	Interferon beta-1a, Teriflunomide, Fingolimod, Rituximab, Ocrelizumab
**15**	Interferon beta-1a, Fingolimod, Dimethyl fumarate, Ocrelizumab
**16**	Interferon beta-1b, Fingolimod, Ocrelizumab
**19**	Dimethyl fumarate
**22**	Interferon beta-1a, Ocrelizumab
**24**	Interferon beta-1a, Natalizumab, Ocrelizumab
**29**	Fingolimod
**36**	Dimethyl fumarate
**46**	Teriflunomide
**48**	Interferon beta-1a, Dimethyl fumarate, Fingolimod
**52**	Interferon beta-1a, Dimethyl fumarate
**53**	Interferon beta-1b, Interferon beta-1a, Dimethyl fumarate, Teriflunomide, Ocrelizumab
**59**	Glatiramer acetate, Fingolimod
**61**	Fingolimod, Rituximab
**64**	Natalizumab
**67**	Fingolimod, Natalizumab
**72**	Glatiramer acetate, Dimethyl fumarate, Ocrelizumab
**78**	Interferon beta-1a, Dimethyl fumarate, Ocrelizumab
**79**	Glatiramer acetate
**80**	Interferon beta-1a, Glatiramer acetate, Dimethyl fumarate, Fingolimod
**81**	Fingolimod, Ocrelizumab
**83**	Dimethyl fumarate
**87**	Peginterferon beta-1a, Teriflunomide
**88**	Interferon beta-1a, Peginterferon beta-1a, Dimethyl fumarate, Fingolimod, Natalizumab
**92**	Interferon beta-1b, Dimethyl fumarate, Fingolimod, Natalizumab, Ocrelizumab
**95**	Glatiramer acetate, Dimethyl fumarate
**96**	Natalizumab, Ocrelizumab
**97**	Interferon beta-1b, Dimethyl fumarate
**98**	Glatiramer acetate, Natalizumab
**104**	Peginterferon beta-1a, Dimethyl fumarate, Teriflunomide, Fingolimod, Ocrelizumab
**106**	Interferon beta-1b, Natalizumab
**109**	Fingolimod
**110**	Fingolimod
**111**	Fingolimod
**112**	Interferon beta-1b, Interferon beta-1a, Fingolimod
**113**	Interferon beta-1a, Peginterferon beta-1a, Dimethyl fumarate, Ozanimod
**114**	Dimethyl fumarate, Rituximab
**117**	Peginterferon beta-1a, Ocrelizumab
**118**	Teriflunomide, Rituximab
**120**	Dimethyl fumarate
**121**	Peginterferon beta-1a
**127**	Glatiramer acetate, Dimethyl fumarate, Fingolimod, Ocrelizumab
**128**	Glatiramer acetate, Ocrelizumab
**130**	Fingolimod, Ocrelizumab
**134**	Dimethyl fumarate, Teriflunomide, Fingolimod
**136**	Interferon beta-1a, Natalizumab

Control people with relapsing-remitting MS were treated with the indicated disease modifying treatments in the order of appearance between 2013-2023.

Supplementary Table 5 Baseline retinal layer measurements in the aHSCT cohort

Retinal layer	RRMS	SPMS	PPMS	*p*-value
**pRNFL-G, *μm*, mean (SD)**	87.2 (13.3)	86.2 (18.8)	90.3 (8.7)	0.860[a]
**pRNFL-T, *μm*, mean (SD)**	54.6 (10.4)	55.5 (15.7)	61 (19.6)	0.767[a]
**pRNFL-PMB, *μm*, mean (SD)**	39.9 (8.2)	43.4 (12.1)	47.9 (16.8)	0.269[a]
**GCIPL, *μm*, mean (SD)**	75.3 (10.3)	76.3 (16.4)	75.3 (8.8)	0.833[a]
**INL, *μm*, mean (SD)**	38.2 (3.3)	38.3 (3.6)	39.5 (3.6)	0.630[b]

GCIPL ganglion cell-inner plexiform layer, *INL* inner nuclear layer, *PPMS* primary progressive multiple sclerosis, *pRNFL-G* global peripapillary retinal nerve fiber layer, *pRNFL-PMB* papillomacular bundle of the peripapillary retinal nerve fiber layer, *pRNFL-T* temporal peripapillary retinal nerve fiber layer, *RRMS* relapsing-remitting multiple sclerosis, *SD* standard deviation, *SPMS* secondary progressive multiple sclerosis, *μm* micrometer.

RRMS (n = 20), SPMS (n = 7), and PPMS (n = 5), measured at the last imaging timepoint prior to aHSCT (ranging from 42 to 213 days before transplantation). Seven individuals without pre-transplant retinal images were excluded from this analysis.

[a] Kruskal-Wallis test (two-sided). Test statistics were: pRNFL-G, $\chi^2(2)= 0.30$; pRNFL-T, $\chi^2(2)= 0.53$; pRNFL-PMB, $\chi^2(2)= 2.62$; GCIPL, $\chi^2(2)=0.37$.
[b] ANOVA, $F(2,59)= 0.47$.

Supplementary Table 6 Effects of clinical characteristics on retinal thickness in the aHSCT cohort

Retinal layer	Sex (p-value / $\chi^2(1)$)	Age at aHSCT (p-value / $\chi^2(1)$)	Time between disease onset and aHSCT (p-value / $\chi^2(1)$)	ON History (p-value / $\chi^2(1)$)	Estimate for ON history (μm)
**pRNFL-G**	0.807 / 0.06	0.525 / 0.4	0.201 / 1.63	**<0.001[a] / 24.27**	-5.768[b]
**pRNFL-T**	0.304 / 1.06	0.923 / 0.01	0.837 / 0.04	**<0.001[a] / 24.53**	-5.990[b]
**pRNFL-PMB**	0.425 / 0.64	0.808 / 0.06	0.985 / 0.00	**0.022[a] / 5.26**	-2.988[b]
**GCIPL**	0.729 / 0.12	0.483 / 0.49	0.110 / 2.56	**<0.001[a] / 18.92**	-4.779[b]
**INL**	0.868 / 0.03	0.478 / 0.50	0.069 / 3.31	0.865 / 0.03	-0.057[b]

aHSCT autologous hematopoietic stem cell transplantation, *GCIPL* ganglion cell-inner plexiform layer, *INL* inner nuclear layer, ON Optic Neuritis, *PPMS* primary progressive multiple sclerosis, *pRNFL-G* global peripapillary retinal nerve fiber layer, *pRNFL-PMB* papillomacular bundle of the peripapillary retinal nerve fiber layer, *pRNFL-T* temporal peripapillary retinal nerve fiber layer, *RRMS* relapsing-remitting multiple sclerosis, *SPMS* secondary progressive multiple sclerosis, μm micrometer.

[a] bold values represent p-values below the threshold of statistical significance at alpha of 0.05.
[b] Effects of clinical characteristics on retinal thickness measurements in the aHSCT cohort were estimated using Linear Mixed-Effects Models (LMMs) with likelihood ratio tests (LRTs). For each model, a full model including all predictors was compared to reduced models that excluded one predictor at a time to assess the independent effect of each characteristic. The optic neuritis (ON) history estimate represents the mean difference in retinal layer thickness (in micrometers) associated with a positive ON history. Negative values indicate less retinal tissue (thinning) in individuals with a history of ON in the specified retinal layer.

Supplementary Table 7 Estimated yearly retinal atrophy by disease subtype following aHSCT

Retinal layer	RRMS	SPMS	PPMS	*p*-value
**pRNFL-G, $\mu m/y$, mean (SE)**	-1.113 (0.882)	-1.206 (1.158)	-0.823 (0.737)	0.900[a]
**pRNFL-T, $\mu m/y$, mean (SE)**	-0.405 (0.911)	-0.844 (1.195)	-0.304 (0.761)	0.868[a]
**pRNFL-PMB, $\mu m/y$, mean (SE)**	0.074 (0.959)	-0.466 (1.256)	-0.563 (0.801)	0.461[a]
**GCIPL, $\mu m/y$, mean (SE)**	-0.397 (0.866)	-0.550 (1.110)	-1.164 (0.738)	0.920[a]
**INL, $\mu m/y$, mean (SE)**	-0.077 (0.244)	-0.379 (0.313)	-0.1997 (0.208)	0.886[a]

GCIPL ganglion cell-inner plexiform layer, *INL* inner nuclear layer, *PPMS* primary progressive multiple sclerosis, *pRNFL-G* global peripapillary retinal nerve fiber layer, *pRNFL-PMB* papillomacular bundle of the peripapillary retinal nerve fiber layer, *pRNFL-T* temporal peripapillary retinal nerve fiber layer, *RRMS* relapsing-remitting multiple sclerosis, *SE* standard error, *SPMS* secondary progressive multiple sclerosis, *y* year, $\mu m/y$ micrometers per year.

RRMS (n = 23), SPMS (n = 8), and PPMS (n = 8). Negative values in the atrophy rate indicate a continuing reduction in retinal layer thickness, reflecting tissue loss, while positive values suggest an increase in thickness. Estimates were obtained using linear mixed models.

[a] The p-values represent the overall significance of the disease subtype as a predictor of longitudinal thickness changes after aHSCT for each layer. These values were derived using a likelihood ratio test (LRT) to compare two linear mixed-effects models: a full model that included the interaction term between disease subtype and time after transplantation and a reduced model lacking this interaction term. Test statistics were: pRNFL-G, $\chi^2(2) = 0.21$; pRNFL-T, $\chi^2(2) = 0.28$; pRNFL-PMB, $\chi^2(2) = 1.55$; GCIPL, $\chi^2(2) = 0.17$; and INL, $\chi^2(2) = 0.24$.

Supplementary Table 8 Outlier exclusion analyses for the RRMS pre/post retinal atrophy comparisons

Retinal layer	Original Δ (μm/y) / p / $\chi^2(1)$	Outlier measurements n [pre/post]	Affected participants/ eyes	Cleaned Δ / p / $\chi^2(1)$
**pRNFL-G**	0.497 / 0.085 / 2.98	8 [7 / 1]	5 / 6	0.543 / 0.018 / 5.58
**pRNFL-T**	1.220 / <0.001 / 15.97	10 [10 / 0]	5 / 7	1.230 / <0.001 / 24.61
**pRNFL-PMB**	1.590 / <0.001 / 19.18	5 [3 / 2]	3 / 4	1.520 / <0.001 / 22.57
**GCIPL**	0.651 / 0.010 / 6.67	10 [8 / 2]	2 / 3	0.584 / 0.002 / 9.58
**INL**	-0.105 / 0.474 / 0.51	9 [7 / 2]	4 / 5	-0.229 / 0.045 / 4.0

GCIPL ganglion cell-inner plexiform layer, *INL* inner nuclear layer, *n* number, *pRNFL-G* global peripapillary retinal nerve fiber layer, *pRNFL-PMB* papillomacular bundle of the peripapillary retinal nerve fiber layer, *pRNFL-T* temporal peripapillary retinal nerve fiber layer, *RRMS* relapsing-remitting multiple sclerosis, Δ difference, *y* year, *μm/y* micrometers per year.

For each retinal layer, the table summarizes the original post-minus-pre difference in yearly atrophy rate (Δ, μm/year) from the primary RRMS (n=23) linear mixed-effects model, together with the corresponding p-value. Observation-level outliers were defined separately for each layer as OCT measurements with an absolute standardized residual greater than 2 in the fitted model. The table reports the total number of excluded outlying observations, their distribution across the pre- and post-aHSCT periods, the number of affected participants and eyes, and the post-minus-pre estimate after outlier exclusion. Positive Δ values indicate less retinal thinning after aHSCT than before aHSCT, whereas negative values indicate greater thinning after aHSCT.

Supplementary Table 9 Baseline retinal layer thickness in the control and aHSCT relapsing-remitting MS cohorts

Retinal layer	Control relapsing-remitting MS	aHSCT relapsing-remitting MS	*p*-value
**pRNFL-G, *μm*, mean (SD)**	94.38 (13.04)	87.91 (13.96)	**0.007**[a, c]
**pRNFL-T, *μm*, mean (SD)**	65.79 (15.37)	54.52 (11.7)	**<0.001**[b, c]
**pRNFL-PMB, *μm*, mean (SD)**	49.62 (10.86)	40.3 (8.91)	**<0.001**[a, c]
**GCIPL, *μm*, mean (SD)**	84.89 (9.68)	75.07 (12.39)	**<0.001**[a, c]
**INL, *μm*, mean (SD)**	37.46 (2.68)	38.49 (3.15)	0.482[a]

aHSCT autologous hematopoietic stem cell transplantation*, GCIPL* ganglion cell-inner plexiform layer*, INL* inner nuclear layer, *MS* multiple sclerosis, *pRNFL-G* global peripapillary retinal nerve fiber layer, *pRNFL-PMB* papillomacular bundle of the peripapillary retinal nerve fiber layer, *pRNFL-T* temporal peripapillary retinal nerve fiber layer, *SD* standard deviation, *μm* micrometer.

Control RRMS (n = 48), aHSCT RRMS (n = 23). For the aHSCT cohort, baseline refers to the first included post-aHSCT OCT scan, while for the control cohort, baseline refers to the first included OCT scan.

[a] Wilcoxon rank sum test (two-sided). Test statistics were: pRNFL-G, W=2260; pRNFL-PMB, W=2718; GCIPL, W=2605; INL, W=1550.
[b] Student's t-test (two-sided, unpaired), t(120)=4.29.
[c] bold values represent p-values below the threshold of statistical significance at alpha of 0.05.

Supplementary Table 10 Effect of baseline retinal thickness adjustment on estimated atrophy-rate differences between the aHSCT and control cohorts

Layer	Baseline-unadjusted difference (μm/year)	p-value [a]	AIC	Baseline-adjusted difference (μm/year)	p-value [b]	AIC	Interaction -adjusted difference (μm/year)	p-value [c]	AIC	Model fit comparison (p-value)[d]	Model fit comparison (p-value)[e]
**pRNFL-G**	0.897	0.021	4425.2	0.275	0.014	2769	0.266	0.018	2770.8	<0.001	0.579
**pRNFL-T**	0.539	0.175	4289	0.294	0.001	2714.3	0.205	0.033	2694.8	<0.001	<0.001
**pRNFL-PMB**	0.509	0.081	4077	0.200	0.015	3074.5	0.056	0.585	3046	<0.001	<0.001
**GCIPL**	0.629	0.046	4090	0.090	0.085	2291.4	0.119	0.027	2289.4	<0.001	0.046
**INL**	−0.022	0.844	2647.8	−0.008	0.935	2266.5	−0.010	0.910	2268.1	<0.001	0.555

aHSCT autologous hematopoietic stem cell transplantation, *AIC* Akaike information criterion, *GCIPL* ganglion cell-inner plexiform layer, *INL* inner nuclear layer, *pRNFL-G* global peripapillary retinal nerve fiber layer, *pRNFL-PMB* papillomacular bundle of the peripapillary retinal nerve fiber layer, *pRNFL-T* temporal peripapillary retinal nerve fiber layer, *YSO* years since onset, *y* year, *μm/year* micrometers per year.

This table summarizes sensitivity analyses performed to assess whether baseline retinal thickness imbalance confounds the estimated difference in yearly retinal atrophy rates between the aHSCT and control cohorts. "Baseline-unadjusted" refers to the unadjusted linear mixed-effects model without baseline retinal layer thickness as a covariate. "Baseline-adjusted" refers to the model additionally including eye-specific baseline thickness of the corresponding retinal layer as a fixed effect. "Interaction-adjusted" refers to the model additionally including a years-since-onset (YSO) × baseline retinal thickness interaction term to test whether baseline thickness influenced the estimated slope over time. Positive values indicate lower atrophy rates in the aHSCT cohort than in controls, whereas negative values indicate lower atrophy rates in controls. P-values for each model reflect likelihood-ratio tests of the YSO × Cohort interaction term. Additional p-values (model fit comparison) compare nested models to assess whether inclusion of baseline-related terms improved model fit and are reported together with the Akaike information criterion for each model fit.

[a] Likelihood-ratio test p-values from baseline-unadjusted linear mixed-effects models testing the YSO × cohort interaction, corresponding to the between-cohort difference in yearly retinal atrophy rates. Test statistics were: pRNFL-G, $\chi^2(1)=5.29$; pRNFL-T, $\chi^2(1)=1.83$; pRNFL-PMB, $\chi^2(1)=3.05$; GCIPL, $\chi^2(1)=3.98$; INL, $\chi^2(1)=0.04$.
[b] Likelihood-ratio test p-values from baseline-adjusted linear mixed-effects models testing the YSO × cohort interaction, corresponding to the between-cohort difference in yearly retinal atrophy rates after adjustment for baseline retinal thickness. Test statistics were: pRNFL-G, $\chi^2(1)=6.04$; pRNFL-T, $\chi^2(1)=10.76$; pRNFL-PMB, $\chi^2(1)=5.94$; GCIPL, $\chi^2(1)=2.96$; INL, $\chi^2(1)=0.01$.
[c] Likelihood-ratio test p-values from interaction-adjusted linear mixed-effects models testing the YSO × cohort interaction. These models additionally included the YSO × baseline retinal thickness interaction. Test statistics were: pRNFL-G, $\chi^2(1)=5.62$; pRNFL-T, $\chi^2(1)=4.54$; pRNFL-PMB, $\chi^2(1)=0.30$; GCIPL, $\chi^2(1)=4.89$; INL, $\chi^2(1)=0.01$.
[d] Likelihood-ratio test p-values comparing linear mixed-effects models with versus without baseline retinal layer adjustment. Test statistics were: pRNFL-G, $\chi^2(1)=1658.07$; pRNFL-T, $\chi^2(1)=1576.67$; pRNFL-PMB, $\chi^2(1)=1004.52$; GCIPL, $\chi^2(1)=1800.47$; INL, $\chi^2(1)=383.31$.
[e] Likelihood-ratio test p-values comparing linear mixed-effects models with versus without baseline retinal layer × time interaction adjustment. Test statistics were: pRNFL-G, $\chi^2(1)=0.31$; pRNFL-T, $\chi^2(1)=21.59$; pRNFL-PMB, $\chi^2(1)=30.46$; GCIPL, $\chi^2(1)=3.97$; INL, $\chi^2(1)=0.35$.

Supplementary Table 11 Characteristics of the restricted control cohorts used in the years since onset (YSO)-window sensitivity analysis

Window	YSO interval (y)	N participants / eyes	N Imaging sessions	N Macular volume scans / Ring scans	Mean follow -up (y)	Cohort retained, participants/ eyes (%)
**Unrestricted Dataset**	Unrestricted	48 / 77	488	467 / 477	4.7	100 / 100
**100% window**	0.64 to 23.11	44 / 70	419	401/ 409	4.45	91.7 / 90.9
**95% window**	0.97 to 22.29	43 / 68	407	389 / 397	4.43	89.6 / 88.3
**90% window**	1.89 to 19.62	42 / 67	355	338 / 345	3.9	87.5 / 87
**85% window**	2.24 to 19.18	42 / 67	336	319 / 326	3.62	87.5 / 87
**80% window**	2.7 to 18.59	39 / 62	308	291 / 298	3.55	81.2 / 80.5
**75% window**	2.95 to 18.42	39 / 62	296	279 / 286	3.4	81.2 / 80.5
**70% window**	3.34 to 17.77	38 / 60	279	262 / 269	3.21	79.2 / 77.9

YSO years since onset, *N* number, *y* year

To assess whether longer OCT follow-up in controls influenced between-cohort comparisons, control observations were restricted to progressively narrower central windows of the aHSCT years-since-onset (YSO) distribution, while the aHSCT cohort was left unchanged, as shown in Supplementary Figure 6. A central X% YSO window denotes the interval containing the central X% of aHSCT measurements. The table summarizes, for the original unrestricted control dataset and for the 100%, 95%, 90%, 85%, 80%, 75%, and 70% aHSCT-derived YSO windows, the corresponding YSO interval, number of participants, number of eyes, number of imaging sessions, number of macular volume scans, number of ring scans, mean follow-up per participant, and the percentage of control participants and eyes retained relative to the original control cohort.

Supplementary Table 12 Robustness analyses for cumulative link mixed models of post-aHSCT disability progression

Subtype	Layer	N EDSS measurements / participants	Wald OR	Wald p-value (raw / BH-adjusted)	Bootstrap median OR (95% CI)	LOPO OR range	LOPO direction reversal	LOPO significant models (n/N)	Max gradient
**RRMS**	**pRNFL-G**	107 / 19	1.026	0.155 / 0.249	1.021 (0.912 to 1.072)	1.01 to 1.037	No	4/19	0.0686
**RRMS**	**pRNFL-T**	107 / 19	1.040	0.174 / 0.249	1.028 (0.896 to 1.142)	1.013 to 1.063	No	7/19	0.00625
**RRMS**	**pRNFL-PMB**	107 / 19	1.037	0.361 / 0.362	1.022 (0.812 to 1.195)	0.98 to 1.077	Yes	4/19	0.00165
**RRMS**	**GCIPL**	107 / 19	1.012	<0.001/ <0.001	1.006 (0.891 to 1.086)	0.985 to 1.037	Yes	4/19	8.57
**RRMS**	**INL**	107 / 19	0.725	<0.001/ 0.002	0.727 (0.526 to 0.975)	0.666 to 0.764	No	19/19	0.000307
**Progressive MS**	**pRNFL-G**	61 / 12	0.932	0.356 / 0.398	0.986 (0.896 to 1.15)	0.967 to 1.016	Yes	7/12	0.00137
**Progressive MS**	**pRNFL-T**	61 / 12	1.016	0.398 / 0.398	1.021 (0.914 to 1.117)	0.98 to 1.033	Yes	4/12	0.00323
**Progressive MS**	**pRNFL-PMB**	61 / 12	1.031	<0.001/ <0.001	1.037 (0.932 to 1.183)	0.996 to 1.062	Yes	4/12	15.8
**Progressive MS**	**GCIPL**	61 / 12	0.982	0.162 / 0.249	0.971 (0.849 to 1.092)	0.96 to 0.985	No	4/12	26.2
**Progressive MS**	**INL**	61 / 12	0.871	<0.001/ <0.001	0.797 (0.333 to 0.927)	0.726 to 0.867	No	10/12	29.8

aHSCT autologous hematopoietic stem cell transplantation, *BH* Benjamini–Hochberg, *CI* confidence interval, *CLMM* cumulative link mixed model, *EDSS* Expanded Disability Status Scale, *GCIPL* ganglion cell-inner plexiform layer, *INL* inner nuclear layer, *LOPO* leave-one-patient-out, *MS* multiple sclerosis, *n/N* number out of, *OCT* optical coherence tomography, *OR* odds ratio, p*RNFL-G* global peripapillary retinal nerve fiber layer, *pRNFL-T* temporal peripapillary retinal nerve fiber layer, *pRNFL-PMB* papillomacular bundle peripapillary retinal nerve fiber layer, RRMS relapsing-remitting multiple sclerosis.

For each subtype and retinal layer, the table summarizes the association between baseline optical coherence tomography (OCT) thickness and longitudinal Expanded Disability Status Scale (EDSS) progression in the post-autologous hematopoietic stem cell transplantation (aHSCT) cumulative link mixed models (CLMM). Reported values include the number of follow-up EDSS measurements and participants for each analysis, the nominal Wald odds ratio (OR), the corresponding Wald p-value, the corresponding Benjamini–Hochberg (BH)-adjusted p-value, the patient-level bootstrap median OR with 95% bootstrap confidence interval from 500 bootstrap refits, and leave-one-patient-out (LOPO) sensitivity results. LOPO analyses were performed by refitting the same model after excluding one patient at a time; the table reports the resulting OR range, whether any direction reversal occurred, and the number of LOPO models remaining statistically significant. In addition, the maximum absolute optimizer gradient (Max gradient) is shown as a numerical model diagnostic.
